# Variety of RNAs in Peripheral Blood Cells, Plasma, and Plasma Fractions

**DOI:** 10.1155/2017/7404912

**Published:** 2017-01-03

**Authors:** Anna V. Savelyeva, Elena V. Kuligina, Dmitry N. Bariakin, Vadim V. Kozlov, Elena I. Ryabchikova, Vladimir A. Richter, Dmitry V. Semenov

**Affiliations:** ^1^Institute of Chemical Biology and Fundamental Medicine, Siberian Branch, Russian Academy of Sciences, Lavrentiev Avenue 8, Novosibirsk 630090, Russia; ^2^Novosibirsk Regional Clinical Oncology Dispensary, Plahotnogo Street 2, Novosibirsk 630108, Russia

## Abstract

Human peripheral blood contains RNA in cells and in extracellular membrane vesicles, microvesicles and exosomes, as well as in cell-free ribonucleoproteins. Circulating mRNAs and noncoding RNAs, being internalized, possess the ability to modulate vital processes in recipient cells. In this study, with SOLiD sequencing technology, we performed identification, classification, and quantification of RNAs from blood fractions: cells, plasma, plasma vesicles pelleted at 16,000*g* and 160,000*g*, and vesicle-depleted plasma supernatant of healthy donors and non-small cell lung cancer (NSCLC) patients. It was determined that 16,000*g* blood plasma vesicles were enriched with cell-free mitochondria and with a set of mitochondrial RNAs. The variable RNA set of blood plasma 160,000*g* pellets reflected the prominent contribution of U1, U5, and U6 small nuclear RNAs' fragments and at the same time was characterized by a remarkable depletion of small nucleolar RNAs. Besides microRNAs, the variety of fragments of mRNAs and snoRNAs dominated in the set of circulating RNAs differentially expressed in blood fractions of NSCLC patients. Taken together, our data emphasize that not only extracellular microRNAs but also circulating fragments of messenger and small nuclear/nucleolar RNAs represent prominent classes of circulating regulatory ncRNAs as well as promising circulating biomarkers for the development of disease diagnostic approaches.

## 1. Introduction

Blood contains RNA within nucleated and enucleated cells as well as cell-free RNA, circulating in membrane vesicles (apoptotic bodies, microvesicles, and exosomes) and also in cell-free ribonucleoproteins. Specific changes in the RNA profile of whole peripheral blood or in the RNA profile of blood fractions such as plasma/serum might reflect physiological and pathological processes occurring in different cells and tissues of the body [1, 2]. Consistently, it was found that peripheral blood cells share more than 80% of the transcriptome with several tissues such as brain, colon, heart, kidney, liver, lung, prostate, spleen, and stomach [3]. Blood is one of the most dynamic tissues, showing both significant intrasubject variation and significant intersubject variation [4]. So, even the whole blood RNA level demonstrates a 3.4-fold range of interindividual difference attributed to differences in cell number and the amount of RNA per cell [5].

Recent studies have concentrated on extracellular RNAs circulating in membrane vesicles such as microvesicles, exosomes, and apoptotic bodies. Microvesicles of 50–2000 nm diameter are budded from the outer plasma membrane of progenitor cells, while exosomes have a smaller diameter of ~30–150 nm and are generated during the maturation of multivesicular bodies (MVB) by multiple invaginations of the late endosome membrane and intraluminal vesicle budding followed by exocytosis of MVB content [1, 6]. Apoptotic bodies have a larger diameter (50–5000 nm) and are released by cells undergoing apoptosis and thereby can contain cell organelles [7, 8].

Most blood cells as well as cells involved in haematopoiesis are able to produce membrane vesicles [1, 9, 10]. Blood plasma is enriched with membrane particles, which are continuously secreted by platelets [1, 11]. Platelets are thought to be a major blood reservoir of membrane vesicles which introduce mRNAs, microRNAs, and other noncoding RNAs (ncRNAs) [12] for capture by other committed recipient cells. Interestingly, platelets are able to release functional mitochondria in a cell-free form enclosed by platelet membrane and, thus, appear as a source of circulating mitochondrial nucleic acids as well [13].

Besides membrane vesicles, blood plasma contains cell-free ribonucleoproteins (RNPs) such as the microRNA/AGO2 complexes [14, 15]. Alongside this, cell-free complexes of microRNAs and high-density lipoproteins (HDL), a component of the reverse cholesterol transport pathway, have been proposed as intercellular mediators of ncRNA communication pathways [16].

RNP complexes of blood plasma have been shown to contain microRNAs [14, 15], while membrane vesicles also contain mRNAs and other cellular RNAs: rRNAs, tRNAs, and long and small noncoding regulatory RNAs [1, 17, 18].

While the most recent studies have focused on the RNA content of purified exosomes, microvesicles, and RNPs, the overall distribution of particular RNA classes and species in the major blood fractions, such as cells, plasma, and plasma fractions, remains under investigation.

In this study, we analysed the RNA profiles of human blood fractions obtained with sequential centrifugation of whole blood and blood plasma. We separated whole blood into pellets of cells, plasma fractions, 16,000*g* and 160,000*g* plasma pellets, and, finally, vesicle-depleted supernatant. We used SOLiD sequencing technology to perform identification, classification, and quantification of blood fraction RNAs. In order to estimate the variability of blood fraction RNA profiles, we used blood samples of healthy donors and non-small cell lung cancer (NSCLC) patients. RNA sequencing data allowed us to describe the composition of transcripts, outline the differentially distributed RNAs, and detect differentially expressed transcripts in blood fractions of NSCLC patients.

## 2. Materials and Methods

### 2.1. Ethics Statement

Healthy volunteers as well as lung cancer patients of Novosibirsk Regional Oncological Dispensary (Novosibirsk, Russia) provided informed written consent.

The study protocol (blood sample collection) was approved by the Institute of Molecular Biology and Biophysics SB RAMS Ethics Committee in accordance with the Declaration of Helsinki of 1975.

### 2.2. Blood Donors and Patients

The study comprised three healthy individuals (males between 45 and 60 years of age) with no clinical history of malignant neoplastic, autoimmune, or chronic inflammatory diseases, three patients (male) with lung adenocarcinoma (LAC) (T_2*a*_N_*x*_M_1_, T_2*b*_N_3_M_1_, and T_3_N_3_M_0_), and four patients (male) with lung squamous cell carcinoma (LSCC) (T_2-3_N_*x*_M_0_). The age of lung cancer patients was from 50 to 60 years.

### 2.3. Blood Sample Collection

Peripheral blood (4 mL) was obtained from the median cubital vein in an EDTA K_3_ BD Vacutainer (BD, USA). Samples were stored at +4°C and subjected to fractionation no later than 2 h after collection.

### 2.4. Blood Fractionation

Blood fractions were obtained with consecutive centrifugations at 4°C, 1,200*g* (20 min), 16,000*g* (20 min), and 160,000*g* (2 h). Platelet-poor plasma samples (1,200*g* supernatants) with absorbance at 414 nm lower than 2.0 were further processed with the next centrifugation steps. Pellets of blood cells (fraction I), plasma (fraction II), and blood plasma particles pelleted at 16,000*g* and 160,000*g* (fractions III and IV) as well as particle-depleted plasma (160,000*g* supernatant, fraction V) were used for RNA isolation immediately after centrifugation. Aliquots of blood fractions were stored at −70°C for subsequent analysis. For the construction of SOLiD sequencing libraries, we used pooled samples of three healthy individuals, pooled samples of three patients with LAC, and pooled samples of four patients with LSCC. Blood cells and blood plasma samples were pooled separately.

### 2.5. Transmission Electron Microscopy (TEM)

Samples of blood fractions were fixed in 4% paraformaldehyde at 4°C overnight, postfixed in 1% OsO_4_, rinsed, dehydrated in graded ethanol and acetone, and embedded in epon-araldite mixture. Ultrathin sections (65–75 nm) were cut with a Leica EM UC7 ultramicrotome (Leica, Germany) and stained with uranyl acetate and lead citrate. The sections were examined with a JEM 1400 (Jeol, Japan) transmission electron microscope equipped with a Veleta digital camera (Olympus Corporation, Japan). For size analysis, iTEM software (Olympus Corporation, Japan) was used.

### 2.6. Dynamic Light Scattering (DLS) Analysis

Blood fraction pellets were resuspended in 1/100 volume of double filtered PBS (dfPBS). DLS was performed using a Malvern Zetasizer Nano ZS (Malvern Instruments Ltd., UK), equipped with a 633 nm He–Ne laser at *T* = 22°C and detection angle of 173°. For each sample, 11 runs of 10 s were carried out three times. Data were analysed with Zetasizer software v7.11 (Malvern Instruments Ltd., UK).

### 2.7. Flow Cytometry Analysis

Aliquots of blood plasma 16,000*g* and 160,000*g* pellets (fractions III and IV) were stained with mouse anti-human CD3-FITC, CD79a-PerCP-Cy5.5, CD41a-FITC, CD34-APC, and CD63-PE according to the manufacturer's recommendations (eBioscience, USA). Pellets were washed twice with dfPBS and analysed by FACSCanto II (Becton Dickinson, USA). Forward scatter and side scatter (FSC and SSC) PMT voltage settings were adjusted for the detection of blood vesicles/extracellular particles (60–1000 nm) using CST beads (Becton Dickinson, USA) and 60 nm polystyrene beads (Thermo Scientific, USA). PMT voltage settings for the detection of FITC, PE, PerCP-Cy5.5, and APC fluorescence were adjusted using Anti-Mouse Ig, *κ*/Negative Control Compensation Particles Set beads, according to manufacturer's recommendations (Becton Dickinson, USA). The following settings were used for flow cytometry analysis: FSC, 615; SSC, 310; FITC, 548; PE, 466; PerCP-Cy5.5, 505; APC, 300; threshold FSC/SSC, 200/200; compensation PE-FITC, 18; and compensation PerCP-Cy5.5/PE, 15. Gates were set according to unstained samples. Flow cytometry data were analysed with BD FACSDiva software v6.1.3 (Becton Dickinson, USA). Overlaid histograms were created using Flowing software v2.5.1 (Turku University, Finland).

### 2.8. RNA Isolation

RNA was extracted using TRIzol reagent (Life Technologies, USA) according to the manufacturer's instructions. We used 50–150 *μ*g/mL glycogen (Thermo Fisher, USA) as a nucleic acid coprecipitant. RNA was dissolved in DEPC-treated water containing 1 u/*μ*L RiboLock RNase Inhibitor (Thermo Fischer, USA) and stored at −70°C. The RNA concentration was measured by microvolume NanoVue Plus spectrophotometer (GE Healthcare, USA) or by Qubit Fluorometer (Life Technologies, USA) in combination with a Qubit RNA HS Assay Kit (Life Technologies, USA) according to the manufacturers' protocols. The yield and size distribution of RNA were determined with a Bioanalyzer 2100 (Agilent Technologies, USA) using an Agilent RNA 6000 Pico Kit (Agilent Technologies, USA).

### 2.9. SOLiD cDNA Library Construction

cDNA libraries were constructed according to Semenov et al. [23]. Briefly, total RNA isolated from pooled blood fractions I–V was processed with DNase I (Thermo Fisher, USA) and FastAP Alkaline Phosphatase (Thermo Fisher, USA) at 37°C for 17 min. The reaction mixture contained 10 mM Tris pH 8.0 (GERBU, Germany), 2 mM MgCl_2_ (Panreac, Spain), 7 mM DTT (AppliChem, USA), 4 u/*μ*L RiboLock (Thermo Fischer, USA), 0.1 u/*μ*L DNase I, and 0.15 u/*μ*L FastAP Alkaline Phosphatase. 0.1 u/*μ*L of RNase III (Invitrogen, USA) was added and reaction mixture was incubated at 37°C for 3 min. Fragmented RNAs were 5′-phosphorylated with ^32^P-*γ*-ATP (1 МBq, 7000 Ci/mM) and 1 u/*μ*L T4 polynucleotide kinase (Biosan, Novosibirsk, Russia) for 1 h at 25°C. RNAs were purified and size-selected (>19 nt) by 14% PAGE with 6 M urea. DNA libraries were constructed with a SOLiD™ Total RNA-Seq Kit (Life Technologies, USA) according to the manufacturer's instructions. The amplification of cDNA was performed using a SOLiD RNA Barcoding Kit Module 1–16 (Life Technologies, USA). We used cDNA amplification with the following protocol: initial denaturation for 2 min at 95°C; 25–28 cycles of 30 s at 95°C, 30 s at 62°C, and 30 s at 72°C; and a final extension of 7 min at 72°C. PCR products were separated by 10% native PAGE. DNAs in the range of 100–200 bp containing inserts of ≤100 nt in length were eluted from the gel, ethanol precipitated, and used for SOLiD sequencing.

### 2.10. SOLiD Sequencing

Sequencing of cDNA libraries was performed by the SB RAS Genomics Core Facility (Novosibirsk, Russia). Library templates were clonally amplified on SOLiD P1 DNA beads using the SOLiD EZ bead system according to the manufacturer's instructions (Life Technologies, USA). The bead-amplified cDNA libraries were processed on a SOLiD 5500xl platform (Life Technologies, USA) with the steps of ligation and detection allowed to obtain arrays of 50 nt sequencing reads.

### 2.11. Sequencing Data Analysis

Mapping of SOLiD sequencing reads was performed with Bowtie 1.1.0 [24]. To resolve the small RNA inserts within 50 nt color space reads, the csfasta and quality data were mapped by 11 consecutive steps of 4, 3, and 2 nt 3′-end-trimming till 19 nt, followed by merging of intermediate SAM files using SAMtools v1.0 [25]. The mapping pipeline included three consecutive filtering steps with the following sets of reference sequences. Reference set I was assembled with sequences of SOLiD adapters and primers, mitochondrial DNA (NC_012920.1), human genomic repeat consensus sequences from Repbase [26], tRNAs [27], human rRNAs (28S, 18S, 5.8S, and 5S), small nuclear RNAs (snRNAs U1–U12), and small cytoplasmic RNAs (scRNAs hY1–hY5) from NCBI RefSeq. Reference set II was assembled with NM and NR records of NCBI human RefSeq RNAs using custom Perl script with human genome GRCh37/hg19. Sequences that were not aligned to reference set I or II were mapped to human genome reference GRCh37/hg19. For the quantification of reads in terms of relative contribution of RNA classes, we used Bowtie mapping with parameter *k* = 1, which produced only one alignment per read. For the quantification of sequencing data in terms of relative RNA abundance (FPKM), we used Bowtie mapping with parameter *k* = 2, allowing two alignments in the set of reference sequences.

Quantitative data in terms of FPKM were obtained with the Cufflinks v2.2.1 suite of tools using Cuffcompare to perform assembled data analysis and Cuffdiff for differential expression analysis [28]. Cuffdiff differential expression analysis was performed with the quartile library normalization method with the blind or per-condition dispersion method for single replication or multiple replications, respectively. To exclude low abundance mapping data, we used a cut-off of 100 (parameter min-alignment-count 100) unless otherwise indicated in the references to tables.

## 3. Results and Discussion

### 3.1. Blood Fractionation and Initial Characterization of Blood Fractions

We separated whole blood of healthy donors and NSCLC patients with sequential centrifugation into five fractions: blood cells, fraction I; plasma, fraction II; plasma particles pelleted at 16,000*g*, fraction III; plasma particles pelleted at 160,000*g*, fraction IV; and vesicle-depleted supernatant, fraction V (Table 1).

**Figure 1:**
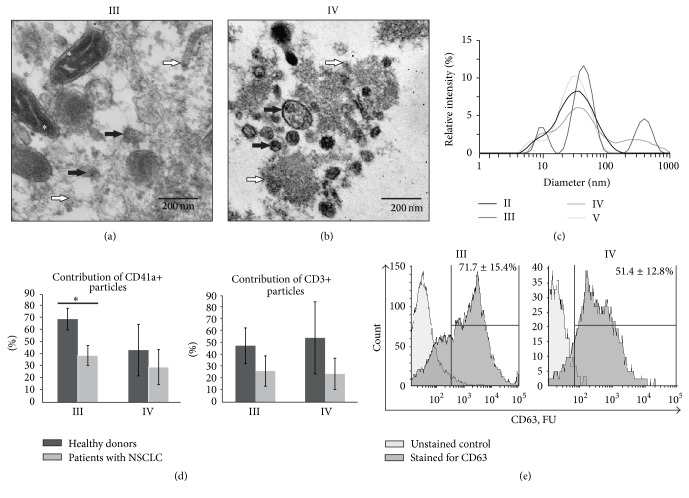
Analysis of intrinsic characteristics of human blood plasma (II) and plasma fractions: particles, pelleted at 16,000*g* (III) and 160,000*g* (IV) and vesicle-depleted plasma (V). ((a) and (b)) TEM analysis of ultrathin sections of fractions III and IV. Black arrows indicate membrane vesicles, white arrows indicate macromolecular aggregates and cell debris, and asterisks indicate mitochondria; (c) DLS analysis of blood fractions II–V; (d) comparative analysis of CD41a+ and CD3+ particle distribution between fractions III and IV of healthy donors and patients with non-small cell lung cancer (NSCLC); (e) flow cytometry assessment of CD63+ particles in fractions III and IV. ^*∗*^*p* < 0.05.

In order to reduce the yield of lysed blood cell components into plasma and plasma fractions, we analysed the plasma haemolysis level according to Kirschner et al. [20]. Only plasma samples with a low haemolysis level were used for subsequent fractionation and examination. Centrifugation followed by ultracentrifugation was applied as a widely used approach for the isolation of extracellular membrane vesicles such as exosomes and microvesicles [29]. We confirmed the presence of membrane vesicles in the 16,000*g* and 160,000*g* pellets of blood plasma (fractions III and IV) by analysis of ultrathin sections of obtained pellets using TEM. It was found that both fractions III and IV were enriched with membrane-covered structures of ~40–100 nm in diameter (Figures 1(a) and 1(b)). Mitochondria and cell debris were also found in blood fraction III (Figure 1(a)) but not in fraction IV (Figure 1(b)).

With the DLS analysis, it was determined that blood plasma and plasma fractions contained two common populations of particles with hydrodynamic diameters of 5–20 nm and 20–100 nm (Figure 1(c)), similar to blood plasma protein complexes (2–70 nm) and small extracellular vesicles such as exosomes (30–100 nm) [7]. At the same time, blood fractions III and IV were distinguished from whole plasma (II) and the vesicle-depleted plasma fraction (V) by the presence of a peak at 150–1000 nm (Figure 1(c)) which is conceivably related to the membrane-covered structures of blood plasma detected by TEM. According to the literature data, a 150–1000 nm peak is mostly related to microvesicles (50–2000 nm), apoptotic bodies (50–5000 nm), or mitochondria (500–3000 nm) [7].

Several types of blood cells release exosomes and microvesicles, which make up the core group of circulating extracellular complexes in human blood plasma [9, 30]. In order to estimate the cellular origin of vesicles pelleted at 16,000*g* and 160,000*g* from blood plasma of healthy donors and lung cancer patients, we used fluorescent monoclonal antibodies to CD3, CD79a, CD41a, and CD34 antigens of T cells, B cells, platelets, and haematopoietic progenitor cells, respectively. It was assessed that 16,000*g* and 160,000*g* particles, pelleted from the plasma of healthy donors, were enriched with the antigens of platelets/megakaryocytes and T cells. Approximately 5% of the 160,000*g* plasma particles were stained with anti-CD79a antibodies to the antigen of B cells. Analysis of the 16,000*g* and 160,000*g* plasma pellets (fractions III and IV) of NSCLC patients revealed a reduced content of CD41a-positive (platelets/megakaryocytes) and CD3-positive (T cells) particles (Figure 1(d)).

Additionally, we analysed the distribution of the commonly used exosome membrane antigen CD63 in membrane particles pelleted at 16,000*g* and 160,000*g* from the blood plasma of healthy donors and patients with NSCLC. It was found that, respectively, 71.7 ± 15.4% and 51.4 ± 12.8% of particles from 16,000*g* and 160,000*g* plasma pellets (blood fractions III and IV) contained CD63 (Figure 1(e)).

Our results, taken together with published data [21, 30], allow us to conclude that blood plasma 16,000*g* and 160,000*g* pellets (fractions III and IV) consist of the composition of circulating particles such as membrane vesicles (exosomes and microvesicles) and macromolecular aggregates secreted by a variety of cells with a prominent contribution of structures derived from platelets/megakaryocytes and T lymphocyte membranes.

### 3.2. Size Distribution of Blood Fraction RNAs

**Figure 2:**
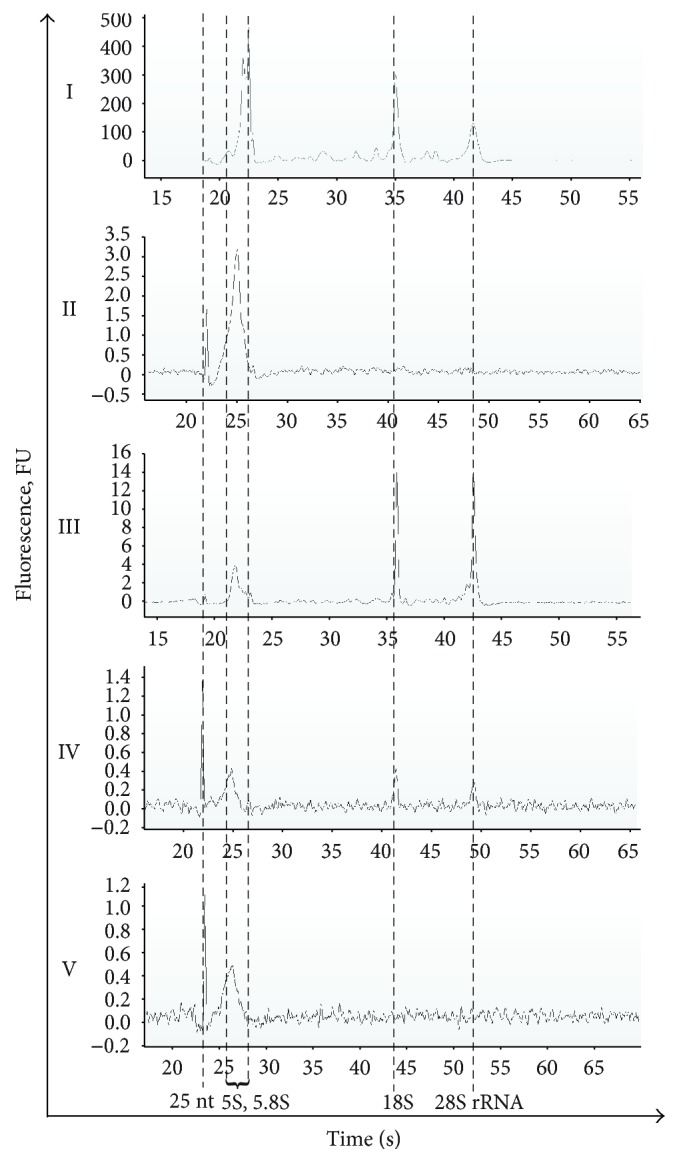
Size distribution of blood fraction RNAs. Total RNA from blood fractions was analysed with an RNA Pico Chip on an Agilent 2100 Bioanalyzer. Data represent the typical profiles for four independent fractionation sets of blood of healthy donors and NSCLC patients.

Total RNA of blood cells (I) and blood vesicles pelleted at 16,000*g* and 160,000*g* (III and IV) contained a prominent amount of full-length 18S and 28S rRNAs. Blood fractions showed the presence of small RNA species and RNA fragments 200 nt and smaller, up to the limitation of fluorescent detection with an Agilent Bioanalyzer (Figure 2). Our data and previously published results [31] indicate that blood plasma fractions contained full-length RNAs and their fragments.

### 3.3. Sequencing of RNA of Human Blood Fractions

To describe the types and the diversity of human blood fraction RNAs, we applied SOLiD high-throughput sequencing technology. We constructed 15 SOLiD cDNA libraries based on fragmented total RNA isolated from five blood fractions of pooled blood samples of healthy donors, of patients with LSCC, and of patients with LAC.

**Figure 3:**
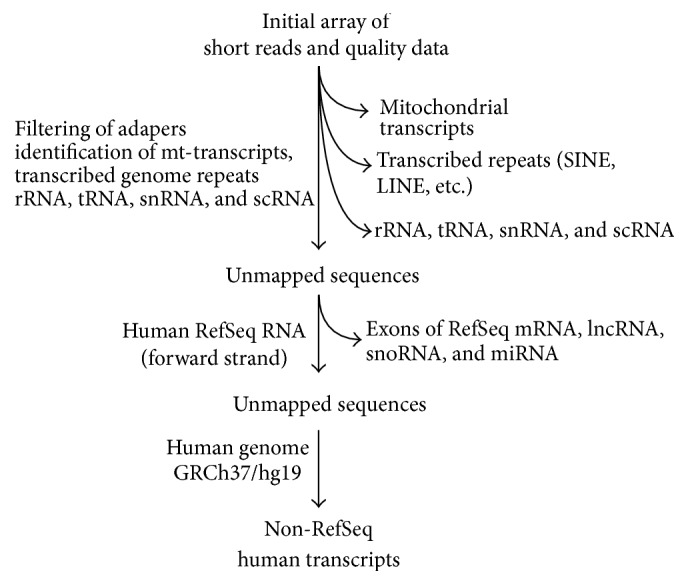
Mapping pipeline.

As the primary result of SOLiD sequencing, we obtained ~9–32 million sequencing reads for 15 cDNA libraries. In order to perform adequate mapping of 50 nt-long reads containing sequences of RNAs that could be as short as 19 nt and flanked by adapter at the 3′-end, we adopted a previously described mapping strategy [23] (Figure 3). Thus, we distinguished the following classes of RNAs: fragments of mitochondrial transcripts; fragments of major cellular RNAs, rRNA, tRNAs, snRNAs, and scRNAs; ambiguous sequences that are related to transcribed human genomic repeats (LINE, SINE, and others); transcripts annotated in the NCBI Human Reference Sequence Database (mRNAs and RefSeq ncRNAs); and non-RefSeq human transcripts (Table 2). As a result, we obtained from ~3 to 15 million mapped reads for each cDNA library.

Assembled sequencing data of healthy donors and of NSCLC patients were analysed with two approaches. First, in order to find commonly and differentially distributed RNAs for a particular blood fraction, we compared RNA profiles of healthy donors, of LSCC patients, and of LAC patients as three independent replicas of the same scheme of blood fractionation. Second, we compared RNA profiles of healthy donors with the RNA profiles of LSCC patients and, separately, with the RNA profiles of LAC patients to outline the disease-associated variations of RNA profiles.

### 3.4. Mitochondrial Transcripts

Blood plasma (fraction II) and blood plasma 16,000*g* pellets (fraction III) were enriched with fragments of mitochondrial RNAs, ~7.7 ± 6.8% and ~10 ± 8.5%, respectively (Table 2). We compared the abundance of individual mitochondrial transcripts in blood fractions, assuming that data of healthy donors and NSCLC patients for each particular blood fraction represented independent replications. It was determined that the 16,000*g* pellets (fractions III) compared with whole plasma (II) contain higher quantity of mt-TRND (mt-tRNA-Asp) fragments. In comparison with vesicle-depleted plasma (fraction V), 16,000*g* pellets were enriched not only with mt-TRND but also with mt-RNR2, mitochondrially encoded 16S rRNA (Table S1 in Supplementary Material available online at https://doi.org/10.1155/2017/7404912).

It is known that the method of obtaining cell-free mitochondria is based on centrifugation of cell lysates at 7,000–8,000*g* [32]. Thus, the enrichment of fraction III (16,000*g* pellets) with mitochondrial transcripts can be explained by the presence of cell-free mitochondria in blood plasma fractions. In accordance with this, mitochondria as whole organelles were detected by TEM in 16,000*g* plasma pellets (blood fraction III, Figure 1(a)) and were not found in plasma 160,000*g* pellets or supernatants.

Next, we searched for common mitochondrial transcripts for particular blood fractions of healthy donors and lung cancer patients. The lists of the top 10 most common mitochondrial transcripts include predominantly mt-tRNAs but not mt-rRNAs or mt-mRNAs. mt-tRNA-Met was defined in all top 10 of all analysed human blood fractions (Table S2).

Previously, it has been shown that human blood platelets release respiratory-competent mitochondria both within platelet microvesicles and as free organelles [12, 13]. Our data together with published results indicate that 16,000*g* plasma pellets contain not only blood extracellular vesicles and RNPs but also cell-free mitochondria and as consequence contain higher amount of mitochondrial transcripts, two of which, 16S mt-rRNA and mt-tRNA-Asp, are strongly associated with the fraction.

### 3.5. Fragments of Major Cellular RNAs and Transcribed Genomic Repeats

Sequences aligned to transcribed genomic repeats of L1 (LINE-1) retrotransposons were equally distributed in all blood fractions, with minor variations in L1 subfamilies (Table S3). Such uniform distribution between blood plasma fractions was determined only for L1 transcripts and allows us to suppose that L1 sequence motifs are not involved in the process of RNA loading into extracellular complexes.

The set of major cellular RNAs, commonly distributed in similar blood fractions of healthy donors and lung cancer patients, included a variety of rRNAs, tRNAs, U snRNAs, and Y scRNAs (Table S4). When we compared the distribution of fragments of major cellular RNAs within blood fractions, we found that particles pelleted at 160,000*g* (fraction IV) were enriched with fragments of 28S rRNA and MER41A transcribed genomic repeat (the ERV1 family member of LTR repeats), in contrast with cells (fractions I), whole plasma (fractions II), and vesicle-depleted plasma (fractions V) (Table S1). rDNA transcripts complementary to 28S rRNA (c_28S_rRNA) were significantly overrepresented in vesicle-depleted plasma supernatant when compared with all other blood fractions.

The most prominent enrichment of 160,000*g* pellets was detected for snRNA species. It was found that U1, U5A/B, and U6 snRNA fragments were significantly overrepresented in plasma particles pelleted at 160,000*g* (fraction IV) when compared with whole plasma or with 16,000*g* pellets (Table S1).

Taken together with the data about the enrichment of 160,000*g* plasma pellets with membrane vesicles, our results allow us to suppose that human blood vesicles such as microvesicles or exosomes are enriched with a set of 28S rRNA fragments and snRNAs, U1, U5A/B, and U6.

### 3.6. Fragments of Exons of RefSeq mRNAs and lncRNAs

In order to evaluate the relative distribution of mRNA fragments (NM records in RefSeq) in human blood fractions, we assembled lists of translated pol II transcripts, which showed that the relative abundance in blood fractions of lung cancer patients was comparable with similar blood fractions of healthy donors (Table S5). It was determined that fragments of PRKCH mRNA (encoding protein kinase C-eta) were represented in the list of the top 10 mRNAs of all blood fractions except fraction IV (160,000*g* pellets). Fragments of major histocompatibility complex classes I A and E mRNAs, HLA-A and HLA-E transcripts, were represented in blood cells (I), plasma (II), and 16,000*g* pellets (Table S5). However, despite some overlapping, sets of mRNA species were similarly distributed in blood fractions of healthy donors and lung cancer patients, demonstrating significant differences between blood fractions (Table S5).

To describe functional mRNA groups, we used PANTHER [33] analysis of gene annotations. It was found that mRNAs of blood cells, plasma, 16,000*g* pellets, and 160,000*g* pellets were enriched with histone gene products involved in the process of chromatin assembly and chromatin organization (GO:0031497 and GO:0006325, respectively, with Bonferroni-corrected *p* < 2.2*E* − 05). Additionally, the set of blood cell mRNAs was enriched with gene products involved in the regulation of macrophage-derived foam cell differentiation, PRKCH, ABCG1, and NR1H3 (GO:0010743, *p* < 9.67*E* − 03). Interestingly, blood plasma particles pelleted at 160,000*g* were also enriched with mRNAs coding ribosomal proteins involved in translation, nuclear-transcribed mRNA catabolic process/nonsense-mediated decay, rRNA processing (GO:0006412, GO:0000184, and GO:0006364, *p* < 6.0*E* − 07), and other ribosome-related processes. For the set of 133 mRNAs of the 160,000*g* plasma supernatant, we did not find any significant enrichment with respect to Gene Ontology (GO) annotations (*p* < 0.05).

The variety of lncRNA fragments (RefSeq NR records longer than 200 nt) was significantly increased in plasma particles pelleted at 160,000*g*, 20 species, and vesicle-depleted plasma, 13 species (Table S6, fractions IV and V, resp.).

The greater variety of lncRNA species similarly distributed in blood fractions IV and V of healthy donors and lung cancer patients suggests the diversity of lncRNA selection and loading to membrane vesicles and cell-free RNPs.

### 3.7. MicroRNAs of Human Blood Fractions

Among the most abundant microRNAs, MIR103-1/2 were detected in the list of commonly expressed microRNA species of all blood fractions of healthy donors and lung cancer patients (Table S7). MicroRNAs MIR16-1/2, 107, 126, 223, and 451 were represented in at least three of the five blood fractions as invariable components (Table S7). It should be noted that a similar set of microRNAs (MIR451, 103-1/2, 16-1/2, 223, and 126) was represented in the lists of the top 20 most abundant microRNAs of all blood fractions tested (*data not illustrated*).

These data emphasize that the variable set of microRNAs detected in blood cells, plasma, or plasma fractions contained an invariable subset of microRNA species commonly expressed in blood cells and circulating as part of the extracellular vesicles and cell-free RNPs.

All of the microRNAs mentioned above were also detected by Chen et al. in blood cells, plasma, or serum of healthy subjects and NSCLC patients with Solexa sequencing. The presence of MIR16-1/2, 107, 126, 223, and 451 in human blood fractions was confirmed with RT-PCR [34]. Among the set of invariable microRNAs, MIR16, 103, 126, and 223 were previously detected in blood plasma microvesicles, peripheral blood mononuclear cells (PBMC), or blood platelets [30]. MicroRNAs MIR16, 126, and 223 were found in the top 30 of the exosome fraction and the same three and MIR451 in the top 30 of the HDL fraction of healthy donor blood [16].

Recently, MIR16, 103, and 107 were detected with microarray data analysis, and MIR451, 223, and 16 with qRT-PCR were described as the most abundant microRNA species of microvesicles released by human platelets [12, 35].

Taken together, the literature data and our results support the concept [35] that platelets are thought to be a major blood reservoir of circulating RNAs. Aside from this, our data indicate that not only platelets but also other cells, including lymphocytes, are responsible for the variability of microRNA content in human blood plasma and plasma fractions.

### 3.8. snoRNAs of Human Blood Fractions

Box H/ACA and box C/D small nucleolar RNAs (snoRNAs) represent a class of regulatory ncRNAs which is poorly described with regard to its abundance and relative distribution in blood fractions and its extracellular functions.

The list of snoRNAs, of which fragments were abundant and commonly represented in the fraction of blood cells of healthy donors and NSCLC patients, included 11 snoRNA species. Fraction IV (160,000*g* blood plasma pellets), enriched with membrane vesicles, contained 17 invariable snoRNA species. In contrast with this, whole plasma, 16,000*g* plasma pellets, and 160,000*g* supernatant were together represented by only 16 snoRNA species (Table S8). Fragments of snoRNA84 (NR_003065) were detected in blood cells, in plasma, and in 160,000*g* pellets. Except for this, other snoRNAs were detected in a fraction-specific manner (Table S8).

Therefore, blood plasma particles pelleted at 160,000*g* demonstrated the widest list of snoRNA species invariably distributed between healthy donors and lung cancer patients that did not resemble the sets of blood cell and plasma snoRNAs and included both box C/D and box H/ACA RNAs.

### 3.9. mRNAs, lncRNAs, microRNAs, and snoRNAs Differentially Distributed in Blood Cells, Plasma, and Plasma Fractions

In order to characterize fraction-specific RNA classes, we used the sequencing data of each particular blood fraction as independent replications of the same isolation conditions and performed fraction-to-fraction comparisons of RNA profiles with the Cuffdiff program. We determined that box C/D and box H/ACA snoRNAs represented the most prominent group of blood RNAs, with variable distribution between blood fractions I and V (Table S9). As could be expected, blood cells (fraction I) were enriched with a set of snoRNAs, such as SNORD110, SNORD65, and SNORA31, when compared with blood fractions II–V. In particular, the FPKM log_2_ level of SNORD110 was four to six times higher in blood cells than in plasma, 160,000*g* plasma vesicles, and plasma vesicle-depleted supernatant (Table S9).

With the snoRNA depletion, 16,000*g* and 160,000*g* plasma vesicles demonstrated enrichment with a particular set of mRNAs and microRNAs. Thus, plasma pellets with membrane vesicles preferentially carry microRNA and mRNA cargo.

### 3.10. Fragments of Non-RefSeq RNAs

Experimental reads that failed to align on human RefSeq RNA references were mapped to human genome reference sequences (hg19). Thus, fragments of introns of mRNAs and lncRNAs as well as novel human transcripts were identified (Table S10). For example, plasma RNA encoded in chr3:857189–857263 represents a fragment of the intron of the long intergenic non-protein-coding RNA 1266 (LINC01266). The abundant RNA fragments commonly represented in particles pelleted at 16,000*g* were encoded in chr9:131641128–131641163 and related to a fragment of the pre-mRNA intron of kynurenine aminotransferase 1 (KYAT1). Locus chr13:29401239–29401304 encodes RNA commonly detected in 160,000*g* plasma vesicles, but currently for the locus there is not any gene annotation in UCSC Genes, RefSeq Genes, or ENCODE/GENCODE data sets.

Generally, blood cells, plasma, and plasma fractions contain a specific profile of non-RefSeq RNA fragments (Table S10). However the cellular and extracellular regulatory functions of their RNA precursors are yet to be elucidated.

### 3.11. Differently Expressed Transcripts of Lung Cancer Patients

To outline the transcripts that differentiated blood fractions of NSCLC patients, we performed fraction-by-fraction Cuffdiff comparisons of RNA profiles of healthy donors and LSCC patients as well as of healthy donors and LAC patients (Tables S11 and S12, resp.).

It was determined that both sets of differentially expressed transcripts were revealed for blood fractions of LSCC patients (Table S11) as well as for blood fractions of LAC patients (Table S12), represented by stand-alone fragments of mitochondrially encoded transcripts and transcribed genomic repeats as well as by variety of fragments of small cytoplasmic vault RNAs, mRNAs, lncRNAs, scaRNA, microRNAs, and snoRNAs.

For LSCC patients, lists of differentially expressed transcripts were represented predominantly by fragments of mRNAs in the blood cells, plasma, and 16,000*g* plasma pellets. In RNA sets of 160,000*g* blood plasma pellets (fraction IV) of the LSCC patients and healthy donors, we did not detect any statistically significant differences by Cuffdiff program (Table S11).

However, for the LAC patients, lists of differentially expressed transcripts were enriched with fragments of microRNA and snoRNA species. For example, the list of upexpressed and downexpressed transcripts revealed for blood plasma 160,000*g* supernatant consisted of 21 microRNAs, 38 snoRNAs, and only 14 mRNAs/lncRNAs (Table S12).

It is known that an aberrant level of circulating microRNAs could correlate with pathological processes such as malignant transformation and cancer progression [36, 37]. Therefore, blood plasma microRNAs are considered as a promising class of circulating biomarkers. Together with our data, it can be concluded that not only extracellular microRNAs but also the profile of circulating fragments of mRNA and snoRNAs reflect disease-associated changes.

## 4. Conclusion

In this study, we analysed the RNA profiles of human blood fractions obtained with sequential centrifugation of whole blood and blood plasma of healthy donors and NSCLC patients. With SOLiD sequencing technology, we performed identification, classification, and quantification of RNAs from blood cells, plasma, plasma vesicles and macromolecular complexes pelleted at 16,000*g* and 160,000*g*, and vesicle-depleted supernatant.

Altogether, the results of DLS, TEM, and flow cytometry analysis of blood plasma particles pelleted at 16,000*g* and 160,000*g* showed that both plasma pellets were enriched with a mixture of circulating particles such as exosomes and microvesicles secreted by a variety of blood cells, with a prominent contribution of membrane structures released by platelets/megakaryocytes and T lymphocytes. Additionally, TEM analysis revealed the presence of cell-free mitochondria in 16,000*g* blood plasma pellets.

SOLiD sequencing allowed us to determine cellular RNA species which had similar and distinct distribution patterns in blood fractions of healthy donors and NSCLC patients.

The most intriguing finding was the enrichment of 160,000*g* plasma pellets with ncRNAs generally localized in the nucleus, snRNAs (U1, U5, and U6), and the simultaneous depletion of snoRNAs, both box C/D and box H/ACA RNA species. It was also shown that blood plasma particles pelleted at 160,000*g* were enriched with a set of mRNAs and microRNAs which did not resemble the set of such RNAs of blood cells.

Small RNAs, processed from snRNAs and snoRNAs, as well as rRNAs and tRNAs, have been found recently and proposed as novel regulators of gene expression [37, 38]. Overall, our data show that blood extracellular circulating complexes contain a variable set of RNAs with a prominent contribution of fragments of snRNAs, which might be involved in regulation of vital cellular processes in recipient cells.

Taken together, our data emphasize that blood cells, plasma, and plasma fractions, both enriched or depleted with membrane vesicles, such as exosomes and microvesicles, contain distinctive sets of cellular transcripts and their fragments which vary significantly due to physiological and pathological processes occurring in different cells and tissues of the body. The profile not only of extracellular microRNAs but also of other circulating small RNAs, especially fragments of mRNAs and snoRNAs, could be used for the development of disease diagnostics approaches.

## Supplementary Material

The supplementary material contains 12 tables representing the results of comparative analysis of RNA content of human blood fractions: blood cells, plasma, membrane particles, pelleted at 16,000 g and 160,000 g, and vesicle-depleted supernatant 160,000 g. Tables S1 and S9 contain the data on RNA species differentially distributed between human blood fractions. Tables S2-S8 and S10 contain the data on RNA species with common distribution within particular human blood fractions. Tables S11 and S12 c ontain the data on RNA species differentially expressed in blood fractions of healthy donors and non-small cell lung cancer patients.

## Figures and Tables

**Table 1:** Human blood fractions obtained in the investigation.

Blood fraction	Description	Method of isolation	Characteristics of the method
(I) cells	Whole blood, enriched with blood cells	1,200*g*^*∗*^, pellet	Preparation of platelet-poor plasma [19] and estimation of haemolysis [20]
(II) plasma	Platelet-poor blood plasma	1,200*g*^*∗*^, supernatant	
(III) 16,000*g* plasma pellets	Intermediate fraction containing membrane vesicles and cell debris	16,000*g*^*∗*^, pellet	Isolation of microvesicles [21]
(IV) 160,000*g* plasma pellets	Fraction enriched with exosomes	160,000*g*^*∗∗*^, pellet	High-recovery isolation of exosomes [22]
(V) 160,000*g* plasma supernatant	Vesicle-depleted plasma	160,000*g*^*∗∗*^, supernatant	

^*∗*^Centrifugation for 20 min at 4°C.

^*∗∗*^Centrifugation for 120 min at 4°C.

**Table 2:** Relative contribution of experimental sequences to RNA classes in human blood cells (I), plasma (II), and fractions of blood plasma (III–V) of healthy donors and lung cancer patients. For each experimental read, a single position in the references was allowed.

Fractions^(1)^	(I) Blood cells			(II) Plasma			(III) 16,000*g* pellets			(IV) 160,000*g* pellets			(V) 160,000*g* supernatant		
Donors or patients^(2)^	H	LSCC	LAC	H	LSCC	LAC	H	LSCC	LAC	H	LSCC	LAC	H	LSCC	LAC
Relative contribution (%)															

Mitochondrial RNAs	2.45	5.38	0.72	13.76	8.89	0.33	17.84	12.01	1.01	2.83	1.16	0.12	7.52	0.61	0.09
rRNA	23.69	18.21	27.63	35.01	18.06	4.36	40.39	19.89	1.81	20.60	25.17	51.27	29.61	12.18	2.20
tRNA	0.70	0.36	2.74	2.39	0.70	0.21	0.42	0.23	1.05	0.50	0.57	0.52	1.06	0.86	0.53
YRNA	1.83	0.88	1.53	1.48	1.31	0.37	5.01	1.29	4.80	1.31	0.79	0.64	1.62	0.25	3.73
U1–U17 snRNA and 7SK RNA	0.52	0.22	0.56	0.20	0.07	0.07	0.06	0.04	0.02	0.17	0.31	0.70	0.18	0.15	0.12
SINE^(3)^	1.18	0.27	0.79	0.61	0.28	0.16	0.64	0.47	0.08	0.17	0.26	0.14	0.51	0.13	0.06
LINE^(3)^	0.46	0.23	0.17	0.23	0.35	0.15	0.15	0.28	0.14	0.18	0.32	0.17	0.28	0.51	0.18
LTR^(3)^	0.09	0.06	0.04	0.06	0.09	0.02	0.04	0.07	0.05	0.03	0.10	0.08	0.07	0.17	0.05
DNA^(3)^	0.04	0.08	0.17	0.03	0.05	0.02	0.02	0.05	0.04	0.08	0.06	0.08	0.05	0.06	0.03
Other transcribed repeats^(4)^	0.03	0.07	0.03	0.09	0.07	0.02	0.06	0.05	0.03	0.05	0.25	0.13	0.75	3.85	0.03
mRNA^(5)^	22.50	30.54	26.08	19.80	34.12	30.24	15.21	29.88	31.28	25.16	33.98	20.11	21.09	35.80	31.36
ncRNA^(6)^	22.71	3.55	2.95	2.84	2.92	4.16	1.62	3.27	16.30	2.25	3.13	2.95	3.60	3.19	17.93
Non-RefSeq RNA^(7)^	23.79	40.14	36.60	23.50	33.10	59.89	18.54	32.47	43.39	46.67	33.89	23.08	33.67	42.23	43.69

Number of reads^(8)^															

Total^(8)^	8.0*E*6	2.8*E*6	4.1*E*6	6.0*E*6	4.3*E*6	3.5*E*5	5.3*E*6	5.3*E*6	1.5*E*6	3.6*E*6	4.0*E*6	4.1*E*6	6.2*E*6	3.2*E*6	2.1*E*6

^(1)^Blood fractions according to Table 1.

^(2)^Pooled blood samples of healthy donors (H), patients with lung squamous cell carcinoma (LSCC), and patients with lung adenocarcinoma (LAC).

^(3)^Reads mapped on referenced sequences of SINE-, LINE-, LTR-, and DNA-repeat families from GIRI Repbase. SINE also includes sequences aligned to 7SL RNA (NR_002715).

^(4)^Includes satellites and simple and other repeats from GIRI Repbase.

^(5)^Fragments mapped to mature mRNAs referenced as exons of NM records of NCBI human RefSeq RNA.

^(6)^Fragments of exons of NR records of human RefSeq RNA.

^(7)^Reads mapped to human genome sequences include introns of NM/NR human RefSeq RNAs as well as fragments of novel lncRNAs not annotated in the human RefSeq RNA database.

^(8)^Total number of aligned reads.
