# Supplementary material for: Variety of RNAs in Peripheral Blood Cells, Plasma, and Plasma Fractions
Source: Biomed Res Int. 2017 Jan 3;2017:7404912. doi: 10.1155/2017/7404912 (PMC5239830; doi:10.1155/2017/7404912)
Supplement: Supplementary file 1 — The supplementary material contains 12 tables representing the results of comparative analysis of RNA content of human blood fractions: blood cells, plasma, membrane particles, pelleted at 16,000 g and 160,000 g, and vesicle-depleted supernatant 160,000 g. Tables S1 and S9 contain the data on RNA species differentially distributed between human blood fractions. Tables S2-S8 and S10 contain the data on RNA species with common distribution within particular human blood fractions. Tables S11 and S12 c ontain the data on RNA species differentially expressed in blood fractions of healthy donors and non-small cell lung cancer patients. [file 7404912.f1.pdf]

Table S1: Differentially distributed mtRNAs, major cellular RNAs and transcripts of genomic repeats in blood cells (I), plasma (II) and blood plasma fractions (III–V).

Profile 1	Profile 2	Transcript ID	FPKM1	FPKM2	log_2	q[(1)]
I. Blood cells	II. Plasma	mt-TRNP	4.1E6	6.0E5	-2.8	0.05
	III. 16,000 g pellet	18S_rRNA	1.3E5	3.5E5	1.4	0.005
		TRNI-ATC	4.6E3	2.6E5	5.8	0.003
	IV. 160,000 g pellet	28S_rRNA[(2)]	8.4E4	2.4E5	1.5	0.003
		U13_snRNA	3.4E4	2.4E3	-3.8	0.003
		TRNI-ATC	4.6E3	9.5E4	4.4	0.026
		MER41A[(3)]	1.1E3	2.5E4	4.5	0.039
	V. 160,000 g supernatant	mt-RNR2	4.3E5	2.1E5	-1.0	0.02
		TRNI-ATC	4.6E3	3.6E5	6.3	0.003
		c_28S_rRNA[(2)]	3.3E3	1.6E5	5.6	0.039
II. Plasma	III. 16,000 g pellet	mt-TRND	2.6E5	3.1E6		0.01
		18S_rRNA	1.7E5	3.5E5	1.1	0.003
		28S_rRNA	5.8E4	1.5E5	1.4	0.003
	IV. 160,000 g pellet	mt-D-loop	8.5E2	7.9E3	3.2	0.01
		28S_rRNA	5.8E4	2.4E5	2.1	0.003
		U5A_snRNA	1.7E4	1.1E5	2.6	0.038
		U5B_snRNA	1.3E4	1.1E5	3.1	0.003
		U6_snRNA	2.7E4	3.9E5	3.9	0.003
		MER41A[(3)]	9.8E2	2.5E4	4.7	0.039
	V. 160,000 g supernatant	c_28S_rRNA[(2)]	6.1E3	1.6E5	4.7	0.049
III. 16,000 g pellet	IV. 160,000 g pellet	U1_snRNA	5.7E4	5.7E5	3.3	0.003
		U5A_snRNA	1.3E4	1.1E5	3.1	0.015
		U5B_snRNA	1.5E4	1.1E5	2.9	0.045
		28S_rRNA	1.5E5	5.6E4	-1.4	0.003
	V. 160,000 g supernatant	mt-RNR2	6.3E5	2.1E5	-1.6	0.01
		mt-TRND	3.1E6	5.0E5	-2.7	0.05
		c_28S_rRNA[(2)]	6.4E3	1.6E5	4.6	0.049
IV. 160,000 g pellet	V. 160,000 g supernatant	mt-RNR2	4.5E5	2.1E5	-1.1	0.02
		28S_rRNA	2.4E5	5.6E4	-2.1	0.003
		U1_snRNA	5.7E5	6.8E4	-3.1	0.005
		TRNW-TGG	4.7E4	5.0E5	3.4	0.019
		c_28S_rRNA[(2)]	2.3E4	1.6E5	2.8	0.039
		MER41A[(3)]	2.5E4	1.3E3	-4.3	0.039

[(1)] Benjamini–Hochberg-adjusted p-values of blood fraction FPKM value comparisons (Cuffdiff).
[(2)] Transcripts complementary to 28S rRNA were overrepresented in blood fraction V (160,000 g supernatant).
[(3)] MER41A transcribed genomic repeat was overrepresented in blood fraction IV (160,000 g pellets).

Table S2: Top 10 common mitochondrial transcripts of healthy donors and NSCLC patients in cells, plasma and blood plasma fractions.

I. Blood cells				II. Plasma				III. 16,000 g pellet				IV. 160,000 g pellet				V. 160,000 g supernatant			
ID[(1)]	FPKM[(2)]	Std[(2)]	q[(3)]	ID	FPKM	Std	q	ID	FPKM	Std	q	ID	FPKM	Std	q	ID	FPKM	Std	q
TRNV	7.2E7	6.8E6	0.91	TRNM	1.2E7	4.8E6	0.55	TRNM	1.2E8	1.8E8	0.69	TRNS2	3.7E7	1.8E7	0.55	TRNV	9.9E7	7.1E7	0.65
TRNM	4.2E7	2.1E7	0.57	TRNK	9.4E6	5.5E6	0.53	TRNV	6.5E7	3.1E6	0.99	TRNM	2.8E7	8.6E6	0.80	TRNT	4.9E7	3.7E7	0.51
TRNT	1.9E7	5.6E6	0.78	TRNI	2.6E6	1.8E6	0.59	TRNS2	4.7E7	3.6E7	0.69	TRNH	1.5E7	7.0E6	0.61	TRNM	4.0E7	3.1E7	0.54
TRNK	1.1E7	8.3E6	0.50	TRNE	2.1E6	9.3E5	0.70	TRNH	2.4E7	1.1E7	0.76	TRNL2	5.6E6	3.3E6	0.51	TRNH	3.2E7	2.3E7	0.68
TRNI	5.1E6	3.0E6	0.51	TRNW	1.6E6	6.7E5	0.60	TRNL1	1.2E7	5.2E6	0.76	TRNC	4.7E6	1.9E6	0.73	TRNK	3.1E7	2.6E7	0.62
TRNL2	5.1E6	2.8E6	0.57	TRNA	1.1E6	5.5E5	0.51	TRNT	1.2E7	4.0E6	0.81	TRNN	3.2E6	1.4E6	0.69	TRNS2	2.5E7	4.5E6	0.88
TRNC	3.0E6	3.6E5	0.91	TRNP	6.0E5	1.6E5	0.60	TRNK	9.6E6	4.2E6	0.81	TRNE	2.6E6	3.7E5	0.96	TRNL1	1.8E7	1.8E7	0.51
TRNW	2.3E6	1.5E6	0.55	RNR1	1.4E5	5.9E4	0.53	TRNC	5.9E6	4.7E6	0.69	TRNI	2.0E6	4.6E5	0.90	TRNC	1.2E7	7.6E6	0.51
TRNF	1.3E6	9.1E5	0.51	ATP8	8.1E4	1.3E5	0.55	TRNL2	5.6E6	3.9E6	0.81	TRNW	2.0E6	6.1E5	0.74	TRNN	1.1E7	3.5E6	0.72
TRNY	9.5E5	6.1E5	0.78	ND1	6.2E3	3.6E3	0.51	TRNR	5.1E6	3.6E6	0.81	TRNS1	1.4E6	6.5E5	0.51	TRNQ	4.2E6	1.9E6	0.72

**Pearson correlation (*r*)**					
**H:LSCC**	0.97	0.93	0.90	0.93	0.80
**H:LAC**	0.86	0.73	0.41	0.98	0.47
**LSCC:LAC**	0.90	0.86	0.41	0.85	0.48

(1) Gene IDs, according to the NCBI reference sequence NC_012920.1 (*Homo sapiens* mitochondrion genome, revised Cambridge reference sequence). Genes were selected as the overlapping top 10 results of the Cuffdiff comparison with minimal p-values (Cuffdiff).

(2) Mean FPKM values and standard deviation (Std) of mean FPKM values of all individual samples (obtained from healthy donors, lung adenocarcinoma and lung squamous cell carcinoma patients) for each blood fraction. Data are sorted in descending order by mean FPKM values within each blood fraction block.

(3) Minimal q-values as Benjamini–Hochberg-adjusted p-values of FPKM comparisons of healthy donor, lung adenocarcinoma and lung squamous cell carcinoma patient blood samples (Cuffdiff).

Table S3: Transcribed genomic repeats, fragments of which are commonly represented in human blood fractions of healthy donors and NSCLC patients.

Fraction	I. Blood cells		II. Plasma		III. 16,000 g pellet		IV. 160,000 g pellet		V. 160,000 g supernatant	
					Representatives					
LINE	8[1]	L1PB2, L1MA10, L1MA4A, L1MA9, L1MB1, L1ME3A	14	L1, L1HS, L1M3A, L1M3DE, L1MD2, L1PA14, L1PA2	10	L1, L1M1, L1M3A, L1ME4, L1ME, L1PA14	11	L1, L1M2C, L1MA4, L1MA4A, L1MA5, L1ME4A	14	L1, L1M3B, L1MA9, L1ME3, L1PA7, L1MB1
SINE[2]	1	FAM	0	--	0	--	2	AluYb3a2, AluYbc3a	1	FAM
LTR	6	MLT1A2, LTR5, MER11B, MER11C, MLT2A1, MLT2B2	4	LTR10A, MER21A, MSTB, THE1A	9	MER39B, MLT1E, MER4B, MLT2B3, THE1A	4	LTR7, MLT1, MLT1A2, THE1-INTERNAL	0	-
DNA	1	Tigger4a	0	--	0	--	3	Tigger1, Tigger3b, Tigger2	2	Tigger3b, Tigger2
Satellite	0	--	0	--	0	--	0	--	1	ALR10
Others	4	c_U2-2[3], c_TRNA, c_TRNV, c_TRNK	2	c_TRNY, c_5.8S_rRNA	1	c_HY3_scRNA	0	--	2	c_TRNP, c_tRNW

(1) Number of repeats in the list of the top 20 most invariable RNA species (Cuffdiff $p > 0.68$, Benjamini–Hochberg-adjusted p-values $q > 0.84$).
(2) SINE genomic repeats include Alu, FAM, FLAM and FRAM consensus sequences (GIRI RepBase) and 7SL scRNA reference sequence (NR_002715).
(3) Referenced sequences are complementary to major cellular RNAs – rRNAs, tRNAs, U1–U12 snRNAs, 7SK RNA and hY1–hY5 scRNAs.

Table S4: Top 10 major cellular RNA species (rRNAs, tRNAs, U1–U6, U12 and U17 snRNA, Y scRNAs and 7SK RNA) common for the human blood fractions of healthy donors and NSCLC patients.

**I. Blood cells**				**II. Plasma**				**III. 16,000 g pellet**				**IV. 160,000 g pellet**				**V. 160,000 g supernatant**			
**ID[1]**	**FPKM[2]**	**Std[2]**	**q[3]**	**ID**	**FPKM**	**Std**	**q**	**ID**	**FPKM**	**Std**	**q**	**ID**	**FPKM**	**Std**	**q**	**ID**	**FPKM**	**Std**	**q**
5S	1.3E7	5.5E6	0.76	TRNV	5.5E6	2.3E6	0.85	HY4	3.8E7	1.7E7	0.76	TRNQ	4.8E5	2.6E4	0.94	TRND	1.4E7	5.0E6	0.74
HY4	1.2E7	2.8E6	0.87	5S	5.1E6	2.3E6	0.77	TRNG	1.5E6	4.9E5	0.73	TRNG	4.3E5	6.6E4	0.85	TRNK	3.9E6	1.3E6	0.73
TRNV[4]	2.6E6	1.1E6	0.71	TRNG	1.9E6	6.7E5	0.88	HY5	1.0E6	3.5E5	0.73	TRNR	1.7E5	2.1E4	0.88	HY5	3.7E6	1.0E6	0.75
TRNQ	1.3E6	7.0E5	0.71	HY5	1.7E6	8.4E5	0.77	TRNV	6.6E5	2.2E5	0.76	TRNM	1.1E5	2.5E4	0.80	TRNG	2.8E6	2.7E5	0.94
TRNR	4.7E5	2.5E5	0.71	5.8S	1.3E6	5.1E5	0.85	TRNM	1.9E5	5.5E4	0.81	TRNT	1.0E5	1.6E4	0.85	TRNQ	1.3E6	4.0E5	0.73
TRNT	2.5E5	8.3E4	0.83	TRNI	3.8E5	1.5E5	0.85	TRNR	1.5E5	3.2E4	0.85	18S	7.7E4	1.8E4	0.85	TRNM	8.5E5	1.8E4	0.98
TRNM	2.1E5	1.1E5	0.72	TRNP	2.0E5	6.4E4	0.83	TRNT	1.4E5	4.5E4	0.78	TRNA	7.6E4	2.1E4	0.72	TRNI	8.0E5	4.5E5	0.72
U6	1.4E5	4.1E4	0.83	TRNW	1.9E5	2.2E4	0.95	TRNW	4.9E4	2.2E4	0.73	TRNN	3.3E4	4.7E3	0.85	U1	1.4E5	6.8E4	0.72
TRNW	1.3E5	9.3E4	0.71	TRNR	5.6E4	5.3E3	0.97	TRNL	4.3E4	3.5E4	0.73	U5E	7.5E3	2.1E3	0.70	U2	9.9E4	3.9E4	0.73
TRNP	7.2E4	1.1E4	0.92	TRNY	4.6E4	1.7E4	0.80	U2	1.6E4	7.6E3	0.76	7SK	3.3E2	9.9E0	0.96	U5F	1.6E3	7.9E2	0.73

**Pearson correlation (*r*)**					
**H:LSCC**	0.93	0.87	1.00	0.96	0.94
**H:LAC**	0.96	0.98	1.00	0.99	0.93
**LSCC:LAC**	0.80	0.93	1.00	0.95	0.99

(1) RNAs selected as the top maximal FPKM values, filtered with cut-off $q < 0.7$, in the three Cuffdiff comparisons of particular fractions of the blood of healthy donors (H), lung adenocarcinoma (LAC) and lung squamous cell carcinoma (LSCC) patients.
(2) Mean FPKM values and standard deviation (Std) of mean FPKM values of blood samples obtained from healthy donors, lung adenocarcinoma and lung squamous cell carcinoma patients. Data are sorted in descending order by FPKM values within each blood fraction block.

(3) Minimal q-values as Benjamini–Hochberg-adjusted p-values of FPKM comparisons of healthy donor, lung adenocarcinoma and lung squamous cell carcinoma patient blood samples (Cuffdiff).
(4) Within tRNA isoforms the most representative isoform is shown.

Table S5: Top 10 mRNAs commonly represented in human blood fractions of healthy donors and NSCLC patients.

I. Blood cells				II. Plasma				III. 16,000 g pellet				IV. 160,000 g pellet				V. 160,000 g supernatant			
ID[(1)]	FPKM[(2)]	Std[(2)]	q[(3)]	ID	FPKM	Std	q	ID	FPKM	Std	q	ID	FPKM	Std	q	ID	FPKM	Std	q
PRKCH[(4)]	1.5E3	1.4E3	0.55	PRKCH	1.6E2	3.9E1	0.64	PRKCH	8.2E2	7.9E2	0.59	CLIC2	5.0E2	3.3E2	0.51	TRIM41	7.1E2	9.1E2	0.56
ABCG1	9.1E1	2.6E1	0.72	TIGD6	7.9E1	6.2E1	0.50	HLA-A	2.9E1	3.8E0	0.71	MDH1	4.4E2	3.2E2	0.56	PRKCH	4.1E2	4.2E2	0.55
KLRC4	6.7E1	1.3E1	0.59	OAZ1	4.3E1	1.1E1	0.54	PIP4K2A	2.6E1	8.1E0	0.66	RPL10L	4.1E2	1.8E2	0.57	ZNF367	4.1E2	2.1E2	0.65
CLIC2	6.2E1	2.1E1	0.66	HLA-E	3.2E1	8.1E0	0.66	CALCR	2.6E1	8.2E0	0.51	TMEFF2	3.0E2	1.7E2	0.55	UQCRQ	1.2E2	3.6E1	0.70
RPL39	6.0E1	1.1E1	0.56	CHN2[(5)]	2.8E1	9.0E0	0.51	PCMTD1	2.5E1	6.2E0	0.63	PPP1R15B	2.8E2	1.3E2	0.72	C19orf24	7.1E1	1.8E1	0.55
NR1H3[(5)]	5.9E1	1.7E1	0.57	F13A1	2.7E1	9.6E0	0.53	ARPC2	2.4E1	4.9E0	0.57	CHRNB3	2.8E2	1.2E2	0.58	ZFR2	6.8E1	4.1E1	0.51
ZNF131	5.5E1	1.2E1	0.64	EIF1	2.7E1	4.6E0	0.61	FOXR1	2.2E1	3.9E0	0.53	C9orf100	1.9E2	9.9E1	0.57	OBSCN	6.7E1	3.6E1	0.66
CCNDBP1	5.4E1	1.8E1	0.57	HIST1H4H	2.5E1	2.4E0	0.84	HIST1H2BI	2.1E1	2.8E0	0.71	NCOA3	1.7E2	6.2E1	0.69	LRRC56	5.9E1	5.0E0	0.92
HLA-A	4.8E1	1.2E1	0.60	ZNF212	2.4E1	4.1E0	0.74	HIST1H2BD	1.9E1	3.5E0	0.60	UCHL3	1.5E2	4.8E1	0.52	HS3ST6	5.1E1	1.6E1	0.50
HLA-E	4.6E1	2.0E1	0.51	UCHL3	2.3E1	3.9E0	0.59	IGFL3	1.9E1	3.7E0	0.55	NHP2	1.4E2	1.9E1	0.74	MARVELD3	4.7E1	2.0E1	0.55
**Pearson correlation (*r*)**																			
H:LSCC	0.97			0.97				1.00				0.56				0.55			
H:LAC	0.96			0.75				1.00				0.55				0.19			
LSCC:LAC	1.00			0.77				1.00				0.55				0.28			

(1) mRNAs selected as the top 10 by the mean FPKM value within a particular blood fraction.
(2) Mean FPKM values and standard deviation (Std) of mean FPKM values of blood samples obtained from healthy donors, lung adenocarcinoma and lung squamous cell carcinoma patients. Data are sorted in descending order by mean FPKM within each blood fraction block.
(3) Minimal q-values as Benjamini–Hochberg-adjusted p-values of FPKM comparisons of healthy donor, lung adenocarcinoma and lung squamous cell carcinoma patient blood samples (Cuffdiff).
(4) Underlined mRNAs were detected in the top 10 of two or more blood fractions.
(5) For the splice isoforms of the gene the most representative isoform was selected by FPKM value.

Table S6: RefSeq lncRNAs (NR transcripts > 200 nt) of human blood fractions of healthy donors and NSCLC patients.

NR	ID[(1)]	FPKM[(2)]	Std[(2)]	q[(3)]
**I. Blood cells**				
NR_003365	SUGT1P3	2.6E2	1.2E2	0.50
NR_027513	CCNDBP1	5.6E1	1.9E1	0.59
NR_027487	LOC146880	2.3E1	5.8E0	0.57
NR_001434	HLA-H	2.2E1	2.5E0	0.76
**II. Plasma**				
NR_003008	SCARNA5	5.2E1	3.3E0	0.89
NR_003674	KGFLP1	1.9E1	3.2E0	0.73
**III. 16,000 g pellet**				
NR_024058	YWHAE	3.7E1	8.6E0	0.56
**IV. 160,000 g pellet**				
NR_003051	RMRP	3.6E3	8.7E2	0.82
NR_002312	RPPH1	3.0E3	1.5E3	0.52
NR_015376	NCRNA00200	5.3E2	3.9E2	0.53
NR_027336	LOC100130015	4.9E1	1.0E1	0.66
NR_003034	LOC441601	4.6E1	9.4E-01	0.95
NR_027232	NCRNA00107	4.5E1	1.1E1	0.52
NR_001593	RPL18AP3	4.3E1	5.1E-01	0.97
NR_001560	CYCSP52	4.3E1	7.2E0	0.65
NR_003586	EEF1A1P9	4.3E1	9.7E0	0.58
NR_033902	KIAA1967	3.2E1	1.1E1	0.55
NR_003003	SCARNA17	3.1E1	6.5E0	0.54
NR_024391	LOC647946	3.1E1	8.2E0	0.55
NR_026877	MGC2889	3.1E1	6.0E0	0.64
NR_024444	LOC100133985	2.8E1	2.4E0	0.77
NR_029455	LOC440335	2.7E1	6.3E0	0.52
NR_027322	LOC283070	2.5E1	1.8E0	0.85
NR_027097	LOC100271832	2.4E1	9.5E-01	0.89
NR_034140	C12orf33	2.2E1	3.7E0	0.63
NR_024531	RPL23AP7	2.2E1	1.9E0	0.81
NR_026911	RPL21P28	2.1E1	4.4E0	0.52
**V. 160,000 g supernatant**				
NR_003674	KGFLP1	2.7E1	2.8E0	0.86
NR_033425	TSLP	2.5E1	4.8E0	0.68
NR_003670	KGFLP2	2.2E1	2.0E0	0.90
NR_024020	NOL8	2.0E1	9.9E0	0.52
NR_033370	LOC63930	1.9E1	1.6E0	0.88
NR_003505	PPP4R1L	1.8E1	4.4E0	0.59
NR_034010	SNHG8	1.5E1	2.8E0	0.63
NR_033789	SPRNP1	1.3E1	2.5E0	0.57
NR_024618	LOC100129550	1.2E1	2.0E0	0.75
NR_026877	MGC2889	1.2E1	2.4E0	0.56
NR_024567	LOC100130557	1.2E1	3.0E0	0.52
NR_026863	C21orf84	1.2E1	2.9E0	0.51
NR_002837	UBE2MP1	1.1E1	2.3E0	0.55

(1) NCBI RefSeq NR-annotated lncRNAs selected as the top 20 by mean FPKM value within a particular blood fraction.
(2) Mean FPKM values and standard deviation (Std) of mean FPKM values in the array of sequencing reads of blood samples obtained from healthy donors, lung adenocarcinoma and lung squamous cell carcinoma patients. Data are sorted in descending order by mean FPKM within each blood fraction block.
(3) Minimal q-values as Benjamini–Hochberg-adjusted p-values of FPKM comparisons of healthy donor, lung adenocarcinoma and lung squamous cell carcinoma patient blood samples (Cuffdiff).

Table S7: Common microRNAs of human blood fractions of healthy donors and NSCLC patients.

**I. Blood cells**				**II. Plasma**				**III. 16,000 g pellet**				**IV. 160,000 g pellet**				**V. 160,000 g supernatant**			
**ID[1]**	**FPKM[2]**	**Std[2]**	**q[3]**	**ID**	**FPKM**	**Std**	**q**	**ID**	**FPKM**	**Std**	**q**	**ID**	**FPKM**	**Std**	**q**	**ID**	**FPKM**	**Std**	**q**
MIR451[4]	9.8E7	1.6E8	0.35	MIR16-2	6.3E5	7.5E5	0.34	MIR103-1	5.9E6	6.4E6	0.55	MIR451	1.2E7	1.7E7	0.39	MIR451	7.7E7	1.2E8	0.34
MIR16-1	5.2E6	6.7E6	0.33	MIR103-1	5.8E5	2.6E5	0.68	MIR103-2	5.9E6	6.4E6	0.55	MIR103-1	4.2E5	2.5E5	0.37	MIR103-1	1.1E6	9.3E5	0.36
MIR16-2	4.5E6	4.9E6	0.35	MIR103-2	5.7E5	2.6E5	0.68	MIR126	1.2E6	1.7E6	0.45	MIR103-2	4.2E5	2.5E5	0.37	MIR103-2	1.1E6	9.2E5	0.36
MIR103-2	3.8E6	2.9E6	0.66	MIR16-1	5.0E5	4.9E5	0.34	MIR26A1	7.9E5	1.1E6	0.34	MIR223	3.1E5	1.7E5	0.63	MIR223	1.0E6	1.2E6	0.35
MIR103-1	3.8E6	2.9E6	0.66	MIR223	2.6E5	2.2E5	0.63	MIR26A2	6.1E5	8.7E5	0.34	MIR126	2.1E5	9.6E4	0.43	MIR16-1	1.0E6	1.1E6	0.35
MIR126	1.4E6	1.1E6	0.51	MIR107	1.2E5	1.1E5	0.33	MIR24-1	5.7E5	6.7E5	0.36	MIR425	3.7E4	1.5E4	0.45	MIR16-2	9.1E5	1.1E6	0.35
MIR144	1.2E6	2.0E6	0.34	MIR140	1.1E5	1.4E5	0.35	MIR107	5.4E5	6.6E5	0.33	MIR122	2.3E4	8.9E3	0.33	MIR126	5.2E5	4.4E5	0.37
MIR486	7.3E5	6.7E5	0.45	MIR191	9.8E4	1.1E5	0.35	MIRLET7F2	4.8E5	4.2E5	0.34	MIR130B	6.2E3	1.2E3	0.52	MIR19B1	2.6E5	2.6E5	0.34
MIR19A	7.2E5	1.2E6	0.35	MIR20B	1.1E4	1.1E4	0.32	MIR21	3.0E5	2.1E5	0.54	MIR197	5.1E3	1.4E3	0.47	MIR101-1	4.1E4	3.6E4	0.31
MIR107	5.0E5	4.5E5	0.46	MIR23B	1.0E4	2.8E3	0.39	MIR24-2	2.8E5	3.3E5	0.35	MIR30E	4.8E3	1.4E3	0.33	MIR106B	3.9E4	4.2E4	0.31
MIR17	4.0E5	6.2E5	0.34	MIR320C1	3.1E3	2.7E3	0.30	MIR142	2.7E5	4.1E5	0.33	MIR551B	1.9E3	1.9E2	0.77	MIR101-2	3.5E4	3.1E4	0.31
MIR223	3.9E5	4.8E5	0.39	MIR146A	2.9E3	8.3E2	0.42	MIR23A	2.7E5	2.6E5	0.38					MIR221	2.2E4	2.4E4	0.35
MIR185	3.3E5	3.2E5	0.34	MIR199B	1.5E3	3.2E2	0.59	MIRLET7F1	2.6E5	2.4E5	0.32					MIRLET7I	1.4E4	1.3E4	0.33
MIR142	2.7E5	2.6E5	0.33	MIR766	1.1E3	5.4E2	0.31	MIR17	2.0E5	2.9E5	0.31					MIR106A	7.1E3	6.6E3	0.33
MIR29B2	2.5E5	3.9E5	0.31	MIR320B2	5.1E2	2.3E2	0.36	MIR425	1.7E5	2.4E5	0.33					MIR194-1	4.3E3	3.7E3	0.35
MIR29B1	2.4E5	3.8E5	0.31					MIR130A	1.6E5	2.3E5	0.31					MIR96	2.9E3	2.7E3	0.31
MIR130A	1.9E5	3.1E5	0.32					MIR19B1	1.6E5	2.1E5	0.34					MIR744	1.4E3	1.2E3	0.31
MIR320A	3.6E4	1.1E4	0.53					MIR23B	1.4E5	1.6E5	0.35					MIR148B	1.2E3	1.0E3	0.31
MIR339	8.1E3	7.2E3	0.33					MIR192	3.7E4	6.1E4	0.30								
MIR145	2.7E3	2.3E3	0.33																
MIR326	1.4E3	2.1E2	0.67																
**Pearson correlation (*r*)**																			
H:LSCC	0.68			0.68				0.73				0.98				0.99			
H:LAC	0.94			0.91				0.76				0.96				0.99			
LSCC:LAC	0.72			0.47				0.99				0.99				0.97			

(1) List of microRNAs filtered with Cuffdiff q-value > 0.3 and total number of sequencing reads > 50 within a particular blood fraction.
(2) Mean FPKM values and standard deviation (Std) of mean FPKM values of blood samples obtained from healthy donors, lung adenocarcinoma and lung squamous cell carcinoma patients. Data are sorted in descending order by mean FPKM within each blood fraction block.
(3) Minimal q-values as Benjamini–Hochberg-adjusted p-values of FPKM comparisons of healthy donor, lung adenocarcinoma and lung squamous cell carcinoma patient blood samples (Cuffdiff).
(4) Underlined microRNAs were detected in the top of at least three blood fractions.

Table S8: Top invariable box C/D and box H/ACA snoRNAs of human blood fractions.

I. Blood cells				II. Plasma				III. 16,000 g pellet				IV. 160,000 g pellet				V. 160,000 g supernatant			
**ID[1]**	**FPKM[2]**	**Std[2]**	**q[3]**	**ID**	**FPKM**	**Std**	**q**	**ID**	**FPKM**	**Std**	**q**	**ID**	**FPKM**	**Std**	**q**	**ID**	**FPKM**	**Std**	**q**
SNORD34	1.3E6	2.2E6	0.34	SNORD84	2.8E4	1.3E4	0.32	SNORD33	5.1E3	4.5E3	0.31	SNORD84	5.3E4	3.1E4	0.35	SNORD31	3.7E5	6.0E5	0.36
SNORD27	5.1E5	8.6E5	0.33	SNORD80	1.2E4	1.0E4	0.30	SNORA14B	5.7E2	1.9E2	0.42	SNORD38A	2.9E4	1.9E3	0.84	SNORD25	2.5E5	4.4E5	0.35
SNORD84[4]	2.9E5	1.3E5	0.44	SNORD32A	7.6E3	6.6E3	0.33					SNORD100	1.8E4	1.9E3	0.77	SNORD27	2.2E5	3.8E5	0.35
SNORD46	1.5E4	1.4E4	0.33	SNORD21	2.3E3	1.1E3	0.35					SNORD34	1.8E4	5.7E3	0.55	SNORD22	1.1E4	1.0E4	0.34
SNORA54	6.7E3	6.2E3	0.33	SNORD15A	8.0E2	1.2E2	0.66					SNORD22	1.2E4	4.5E3	0.34	SNORD67	1.3E3	2.7E2	0.54
SNORD114	4.8E3	8.2E2	0.57	SNORA75	6.8E2	1.1E2	0.73					SNORD104	8.3E3	3.8E3	0.39	SNORD94	1.2E3	3.3E2	0.39
SNORA61	2.9E3	2.6E3	0.33									SNORD29	5.4E3	8.1E2	0.74	SNORA14A	4.0E2	8.7E1	0.73
SNORA19	2.6E3	6.2E2	0.40									SNORD38B	4.3E3	1.0E3	0.56	SNORD15A	2.2E2	1.3E2	0.30
SNORA62	2.0E3	1.9E3	0.33									SNORD35A	2.3E3	4.1E2	0.63				
SNORA52	1.8E3	1.5E3	0.33									SNORA63	1.2E3	4.0E2	0.37				
SNORA9	7.3E2	6.2E2	0.33									SNORD67	9.8E2	3.7E2	0.32				
												SNORA17	4.7E2	1.9E2	0.30				
												SNORA48	4.6E2	1.3E2	0.51				
												SNORA71A	4.4E2	1.2E2	0.63				
												SNORA57	3.5E2	1.8E2	0.34				
												SNORA6	3.3E2	1.2E2	0.35				
												SNORA53	5.6E1	1.7E1	0.44				
**Pearson correlation (*r*)**																			
H:LSCC	1.00			0.53				--				0.98				0.65			
H:LAC	1.00			0.83				--				0.96				0.85			
LSCC:LAC	0.99			0.91				--				0.99				0.94			

(1) snoRNAs selected as the top 20 by mean FPKM value, filtered with minimal *q* > 0.4, within a particular blood fraction. Selected snoRNAs were represented by more than 100 reads in each cDNA library.

(2) Mean FPKM values and standard deviation (Std) of mean FPKM values of blood samples obtained from healthy donors, lung adenocarcinoma and lung squamous cell carcinoma patients. Data are sorted in descending order by mean FPKM within each blood fraction block.

(3) Minimal q-values as Benjamini–Hochberg-adjusted p-values of FPKM comparisons of healthy donor, lung adenocarcinoma and lung squamous cell carcinoma patient blood samples (Cuffdiff).

(4) SNORD84 represented in the top list of invariable snoRNAs of three blood fractions (blood cells, plasma and 160,000 g pellets of blood plasma).

Table S9: Differentially distributed mRNA, lncRNAs, microRNAs and snoRNAs in blood cells, plasma and plasma fractions.

Profile 1[(1)]	Profile 2[(1)]	Transcript ID	FPKM1	FPKM2	log_2	q[(2)]	Total Number[(3)] NM/miR/sno/others[(4)]
I. Blood cells	II. Plasma	RBBP8[(5)]	1.3E1	2.3E3	7.0	0.04	5 5/0/0/0
		AURKAIP1	5.0E0	3.7E1	3.0	0.04	
		IFNA7	3.0E0	1.7E1	2.0	0.04	
		SNORD110	5.7E4	4.7E3	-4.0	0.04	8 0/3/5/0
		SNORA66	3.4E3	2.7E2	-4.0	0.04	
		MIR210	1.1E4	7.3E2	-4.0	0.04	
	III. 16,000 g pellet	MIR3182	4.2E3	1.6E5	5.2	0.04	12 6/5/0/1
		MIR744	3.7E2	1.2E4	5.0	0.04	
		MIR10A	2.2E2	4.3E3	4.3	0.04	
		SNORA8	3.5E3	1.5E2	-4.5	0.04	39 8/2/23/6
		SNORA7A	3.1E3	1.3E2	-4.6	0.04	
		SNORD94	4.4E3	1.2E2	-5.2	0.04	
	IV. 160,000 g pellet	FAM187B	6.4E0	1.7E2	4.7	0.04	76 68/1/0/7
		KCNIP4	6.4E0	1.0E2	4.0	0.04	
		C2orf7	6.3E0	7.9E1	3.7	0.04	
		SNORD56	8.0E4	9.6E2	-6.4	0.04	82 7/18/49/8
		SNORD65	5.5E4	6.4E2	-6.4	0.04	
		SNORD110	5.7E4	6.7E2	-6.4	0.04	
	V. 160,000 g supernatant	TMEM91	3.7E0	4.7E1	3.7	0.04	28 24/1/0/3
		MIR10A	2.2E2	2.4E3	3.4	0.04	
		OSTN	7.4E0	5.7E1	2.9	0.04	
		SNORD1B	7.8E3	5.0E2	-4.0	0.04	18 6/1/9/2
		SNORA31	3.5E3	1.8E2	-4.3	0.04	
		SNORD110	5.7E4	2.8E3	-4.3	0.04	
II. Plasma	III. 16,000 g pellet	MIR421	1.4E3	2.0E4	3.8	0.04	4 0/4/0/0
		MIR139	3.2E3	3.6E4	3.5	0.04	
		MIR199B	1.5E3	1.5E4	3.2	0.04	
		--	--	--	--	--	0
	IV. 160,000 g pellet	FAM187B	1.4E1	1.7E2	3.6	0.04	17 16/0/0/1
		FAM185A	8.9E0	1.1E2	3.6	0.04	
		PRNP	3.1E0	2.5E1	3.0	0.04	
		C1D	3.2E1	2.5E0	-3.7	0.04	11 9/0/2/0
		METT5D1	3.4E3	3.1E1	-6.8	0.04	
		RBBP8	2.3E3	9.7E0	-7.9	0.04	
	V. 160 000g supernatant	TMEM91	5.4E0	4.0E1	2.9	0.04	5 3/1/0/1
		FLJ41278	3.9E3	2.5E4	2.7	0.04	
		MIR451	1.8E7	9.0E7	2.3	0.04	
		--	--	--	--	--	0
III. 16,000 g pellet	IV. 160,000 g pellet	MIR449B	6.7E2	1.1E4	4.0	0.04	22 17/1/2/2
		RNU11	7.1E2	7.2E3	3.3	0.04	
		KRTAP10-5	1.0E1	9.5E1	3.2	0.04	
		SNORD110	1.7E4	6.7E2	-4.6	0.04	33 2/21/9/1
		MIR744	1.2E4	3.5E2	-5.1	0.04	
		SNORD96A	3.2E4	6.8E2	-5.6	0.04	
	V. 160,000 g supernatant	SNORD94	1.2E2	1.4E3	3.5	0.04	7 4/1/2/0
		SNORD38A	5.5E3	3.5E4	2.7	0.04	
		CHI3L2	3.7E0	1.8E1	2.2	0.04	
		MYL6	7.4E1	1.4E1	-2.4	0.04	1 1/0/0/0
IV. 160,000 g pellet	V. 160,000 g supernatant	C1D	2.5E0	5.1E1	4.4	0.04	15 4/6/5/0
		MIR4306	1.0E3	1.5E4	3.9	0.04	
		SNORD20	7.1E2	9.0E3	3.7	0.04	
		ATP5G2	3.8E1	6.5E0	-2.6	0.04	13 12/0/0/1
		ADAM29	2.3E1	3.4E0	-2.8	0.04	
		CELA2B	4.3E1	4.1E0	-3.4	0.04	

(1) Human RefSeq RNA profiles of blood fractions were compared with Cuffdiff (cutoff 100) and 3 most enriched as well as 3 most depleted RNA species for each pairs of comparisons represented.
(2) Benjamini–Hochberg adjusted *p*-values of blood fractions FPKM values comparisons (CuffDiff).

[3] Total number of differentially distributed RefSeq RNAs for each block of enriched or depleted species.
[4] Total number of mRNAs/microRNAs/snoRNAs/other ncRNAs (including lncRNAs) for each block of enriched or depleted species.
[5] For mRNAs with multiple splice isoforms the most exerted isoform was selected by log_2 value of FPKM comparisons.

Table S10: Top invariable non-RefSeq RNAs of human blood fractions (fragments of pre-mRNA introns, lncRNAs and novel transcripts).

I. Blood cells			II. Plasma			III. 16,000 g pellet			III. 160,000 g pellet			V. 160,000 g supernatant		
Chr[1]	FPKM[2]	Std[2]	Chr	FPKM	Std	Chr	FPKM	Std	Chr	FPKM	Std	Chr	FPKM	Std
chr8[+]12751238-12751281	8.2E7	2.9E7	chrX[+]120612032-120612062	1.8E7	1.1E7	chr4[+]186518885-186518942	6.8E7	3.2E7	chr2[-]28688454-28688543	7.5E8	5.0E8	chr7[+]106008992-106009055	1.9E8	1.2E8
chr4[-]190968942-190968967	6.9E7	2.9E7	chr1[-]8053517-8053562	1.7E7	4.4E6	chr9[-]131641128-131641163	6.2E7	4.1E7	chr13[-]29401239-29401304	2.3E8	1.1E8	chr10[-]129916968-129917004	1.9E8	1.3E8
chr10[-]135460779-135460804	6.4E7	2.6E7	chr3[+]857189-857263	1.7E7	1.0E7	chrX[-]131244850-131244874	6.0E7	3.5E7	chr19[-]24187279-24187313	1.8E8	6.3E7	chr8[-]104001494-104001575	1.8E8	1.1E8
chr2[-]224644694-224644813	4.5E7	1.9E7	chr7[+]34253855-34253942	1.6E7	8.9E6	chrY[-]24224242-24224292	5.5E7	8.0E6	chr17[-]3989516-3989581	1.8E8	1.0E8	chr12[+]123449033-123449056	1.6E8	6.1E7
chr10[-]60691390-60691418	4.3E7	2.5E7	chr12[-]5022311-5022331	1.6E7	5.4E6	chrY[-]25833919-25833969	5.5E7	8.0E6	chr4[+]134994298-134994322	1.6E8	3.9E7	chr12[+]78353818-78353840	1.2E8	5.3E7
chr1[-]183949763-183949807	3.8E7	2.2E7	chr20[-]41004960-41005077	1.2E7	4.8E6	chr21[-]24341920-24341947	4.6E7	1.7E7	chr12[+]78353818-78353840	1.3E8	3.7E7	chr5[-]155785942-155785993	9.2E7	5.3E7
chr16[-]16982934-16982961	3.6E7	1.4E7	chr15[+]58251879-58251992	1.1E7	1.4E6	chr6[-]144210182-144210221	4.4E7	1.4E7	chr18[-]21707456-21707476	1.1E8	7.4E7	chr12[-]126701820-126701923	9.0E7	3.9E7
chr5[-]74063376-74063516	3.5E7	1.5E7	chr1[-]111708389-111708411	1.1E7	5.7E6	chr9[+]129123637-129123704	4.3E7	1.4E7	chr1[-]87222201-87222238	1.1E8	6.0E7	chr6[+]132015523-132015683	8.7E7	1.1E7
chr22[-]36301696-36301718	3.4E7	1.6E7	chr18[-]7745099-7745133	1.1E7	6.6E6	chr3[-]25177183-25177216	3.4E7	1.4E7	chr16[-]32696379-32696407	1.1E8	6.8E7	chr19[+]45361161-45361250	7.7E7	4.7E7
chr1[-]215515108-215515196	3.2E7	1.3E7	chr7[-]65637753-65637775	8.5E6	2.8E6	chr7[-]146153014-146153086	3.0E7	1.9E7	chr13[+]113415433-113415464	1.1E8	2.7E7	chr1[+]193231400-193231565	6.3E7	4.1E7
chr1[-]97294603-97294682	3.1E7	2.1E7	chr15[-]54722229-54722333	8.2E6	5.2E6	chr8[+]12751238-12751281	3.0E7	1.8E7	chr14[+]53235829-53235849	1.0E8	6.3E7	chr3[+]137352004-137352055	5.7E7	6.1E5
chr3[-]115194392-115194518	3.0E7	1.9E7	chr7[-]106045206-106045234	7.1E6	3.6E6	chr4[-]190968942-190968967	3.0E7	1.2E7	chr8[-]104001494-104001575	8.9E7	2.6E7	chr18[-]74718233-74718260	5.6E7	3.4E7
chr3[+]69703070-69703090	2.9E7	1.5E7	chr4[-]145789285-145789371	6.7E6	3.0E6	chr1[+]193231400-193231565	3.0E7	1.9E7	chr3[-]45713728-45713819	8.6E7	3.5E7	chr4[+]5320371-5320402	5.5E7	3.3E7
chr3[+]44188974-44189054	2.7E7	1.8E7	chr7[-]90757495-90757572	6.6E6	4.2E6	chr10[-]135460779-135460804	3.0E7	1.2E7	chr12[+]90388361-90388432	8.6E7	3.2E7	chr6[-]108111274-108111294	5.4E7	1.4E7
chr6[+]75775575-75775595	2.3E7	1.4E7	chr1[+]162406742-162406799	5.9E6	3.7E6	chr1[-]209056366-209056417	2.6E7	9.5E6	chr11[-]81934926-81935013	8.4E7	5.0E7	chr1[-]53369280-53369310	5.0E7	2.2E7
chr11[+]15314015-15314142	2.3E7	1.2E7	chr1[-]11189305-11189372	5.7E6	7.2E5	chr18[+]13870664-13870696	2.6E7	1.7E7	chr10[+]36672457-36672544	8.1E7	5.2E7	chr2[-]118804188-118804241	4.7E7	1.9E7
chr4[-]190591958-190592030	2.2E7	1.3E7	chr4[-]177405455-177405515	5.6E6	3.2E6	chrX[+]134761028-134761050	2.5E7	1.3E7	chr5[-]74063376-74063516	8.1E7	4.3E7	chr5[-]22102694-22102725	4.6E7	1.9E7
chr7[+]95709490-95709583	2.2E7	9.6E6	chr9[-]22206042-22206161	5.5E6	2.1E6	chr21[-]32489655-32489703	2.2E7	1.4E7	chr7[-]42618945-42619083	7.6E7	1.8E7	chr17[-]3989516-3989581	4.1E7	2.4E7
chrY[+]8231760-8231792	1.9E7	9.5E6	chr9[-]109601236-109601333	5.5E6	1.9E6	chr10[-]89537898-89537924	2.2E7	1.4E7	chr3[-]111399254-111399276	7.5E7	4.1E7	chr9[-]131641128-131641163	3.8E7	1.9E7
chr11[+]4499105-4499138	1.9E7	1.3E7	chr8[+]83366132-83366300	5.3E6	3.0E6	chr13[-]40708839-40708878	2.0E7	1.0E7	chr7[-]127077302-127077369	7.2E7	4.0E7	chr16[-]32696379-32696407	3.8E7	8.7E6

[1] Non-RefSeq RNA fragments selected as the top 20 by mean FPKM value, filtered with FPKM to Std ratio > 1.5, within a particular blood fraction.
[2] Mean FPKM values and standard deviation (Std) of mean FPKM values of blood samples obtained from healthy donors, lung adenocarcinoma and lung squamous cell carcinoma patients. Data are sorted in descending order by mean FPKM within each blood fraction block.

Table S11: Top differentially expressed transcripts of blood fractions of lung squamous cell carcinoma patients.

Fraction	Regulation	Transcript ID	FPKM1[(1)] H	FPKM2[(1)] LSCC	log_2	q[(2)]	Total Number[(3)] NM/miR/sno/others[(4)]
I. Blood cells	Up	MIR214	4.4E2	1.3E4	4.9	0.050	38<br>29/7/0/1
		MIR766	6.8E2	1.5E4	4.4	0.020	
		MRPS33	9.1E0	1.3E2	3.9	0.048	
		MIB2 NR_033183	2.0E0	2.7E1	3.8	0.013	
		SNCB[(5)]	5.5E0	7.6E1	3.8	0.038	
		NIT1	3.1E0	3.7E1	3.6	0.043	
		TRAPPC2L	7.4E0	8.9E1	3.6	0.040	
		ARHGAP11B	3.5E0	3.9E1	3.5	0.040	
		HCFC1R1	5.4E0	6.0E1	3.5	0.036	
		MIR197	4.4E3	4.7E4	3.4	0.043	
	Down	IFNA17	5.9E1	3.3E0	-4.2	0.032	97<br>27/21/40/9
		SNORD82	4.8E4	2.3E3	-4.4	0.032	
		SNORD42A	1.2E5	5.5E3	-4.4	0.040	
		IFNA10	6.2E1	2.7E0	-4.5	0.027	
		RPL17	1.8E2	7.7E0	-4.5	0.040	
		C17orf91	1.0E2	3.6E0	-4.8	0.035	
		SNORA31	8.9E3	2.6E2	-5.1	0.044	
		SCARNA9	4.7E2	1.2E1	-5.3	0.046	
		MIR106A	5.6E4	1.3E3	-5.4	0.044	
		C17orf91	1.1E2	2.4E0	-5.5	0.032	
II. Plasma	Up	SLAMF9	7.0E0	5.0E1	2.8	0.038	15<br>10/2/1/2
		TBC1D17	6.9E0	3.2E1	2.2	0.028	
		OVCA2	1.2E1	5.1E1	2.1	0.025	
		SSBP4	6.4E0	2.7E1	2.1	0.029	
		CCNB3	8.5E0	3.5E1	2.0	0.045	
		ACSM3	1.0E1	4.0E1	2.0	0.035	
		GIMAP5	1.1E1	4.6E1	2.0	0.048	
		LOC285074	7.9E0	2.7E1	1.8	0.039	
	Down	MIR26B	4.2E4	1.9E3	-4.5	0.039	147<br>96/23/5/23
		MIR29B2	4.5E4	1.9E3	-4.5	0.033	
		MIR17	1.0E5	4.0E3	-4.7	0.019	
		MIR25	4.4E4	1.7E3	-4.7	0.033	
		NPY	4.2E2	1.3E1	-5.0	0.025	
		MIR15B	6.2E4	1.8E3	-5.1	0.009	
		MIR101-2	6.5E4	1.7E3	-5.2	0.035	
		SNORD34	2.4E5	3.3E3	-6.2	0.032	
		MIR15A	1.2E5	1.5E3	-6.3	0.041	
		MIR451	1.6E7	1.6E5	-6.7	0.009	
III. 16,000 g pellet	Up	SLC26A5	4.5E0	7.4E1	4.0	0.004	94<br>54/24/3/13
		LOC340357	9.6E0	1.0E2	3.4	0.004	
		MIR620	1.2E3	1.1E4	3.2	0.032	
		KDM4C	6.1E0	5.2E1	3.1	0.029	
		LOC284412	8.1E0	6.8E1	3.1	0.024	
		MIR421	3.5E3	2.9E4	3.0	0.005	
		RAB4B	1.5E1	1.0E2	2.8	0.010	
		TXNDC11	8.0E0	5.5E1	2.8	0.028	
		CCNB3	9.0E0	6.1E1	2.8	0.004	
		IL18	5.8E0	3.9E1	2.7	0.017	
	Down	VTRNA1-2	2.6E4	2.1E3	-3.6	0.013	273<br>221/14/7/31
		MIR144	6.3E4	4.9E3	-3.7	0.004	
		MIRLET7A3	1.6E5	1.2E4	-3.7	0.013	
		NCRNA00173	2.0E2	1.4E1	-3.8	0.016	
		SF1	4.2E1	2.8E0	-3.9	0.004	

Fraction	Regulation	Transcript ID	FPKM1[1] H	FPKM2[1] LSCC	log_2	q[2]	Total Number[3] NM/miR/sno/others[4]
		NCRNA00114	1.7E2	1.1E1	-3.9	0.010	
		SNORD31	1.6E5	9.7E3	-4.0	0.004	
		CGB1	2.0E2	1.1E1	-4.1	0.004	
		RPL9	5.2E2	2.9E1	-4.2	0.046	
		CSN3	2.9E2	1.4E1	-4.3	0.004	
**V. 160,000 g supernatant**	**Up**	MIR524	0.0E0	5.2E4	n/d[6]	0.028	1<br>0/1/0/0
	**Down**	EPPK1	1.4E3	1.3E1	-6.8	0.028	13<br>1/3/9/0
		MIR1306	2.0E3	0.0E0	n/d	0.028	
		SNORD103B	3.7E3	0.0E0	n/d	0.028	
		SNORD49A	5.5E3	0.0E0	n/d	0.028	
		SNORD36C	7.6E3	0.0E0	n/d	0.028	
		MIR369	1.3E4	0.0E0	n/d	0.028	
		SNORD28	1.3E4	0.0E0	n/d	0.028	
		MIR3182	3.1E4	0.0E0	n/d	0.028	
		SNORD48	4.8E4	0.0E0	n/d	0.028	

(1) Human RNA profiles of healthy donors and patients with lung squamous cells carcinoma were compared fraction by fractions with Cuffdiff (cutoff 100). The most up regulated as well as the most down regulated RNA species for each pairs of comparisons are represented. Data sorted by log_2 in descending or ascending order for up or down regulated transcripts, respectively.
(2) Benjamini–Hochberg adjusted p-values of blood fractions FPKM values comparisons (CuffDiff). Benjamini–Hochberg-adjusted p-values of blood fractions FPKM values comparisons of healthy donor and lung adenocarcinoma patient blood fractions (Cuffdiff).
(3) Total number of differentially regulated RNAs for each block of up or down regulated species.
(4) Total number of mRNAs/microRNAs/snoRNAs/other ncRNAs (including lncRNAs, mtRNA, transcribed genome repeats rRNAs, tRNAs, U snRNAs, Y scRNAs) for each block of up or down regulated species.
(5) For mRNAs with multiple splice isoforms the most exerted isoform was selected by log_2 value of FPKM comparisons.
(6) For zero FPKM value in one of the compared fraction log2 value not determined (n/d). The data sorted by non zero FPKM value.

Table S12: Top differentially expressed transcripts of blood fractions of lung adenocarcinoma patients.

Fraction	Regulation	Transcript ID	FPKM1[1] H	FPKM2[1] LSCC	log_2	q[2]	Total Number[3] NM/miR/sno/others[4]
I. Blood cells	Up	MIR517A	0.0E0	3.6E3	n/d[5]	0.020	1<br>0/1/0/0
	Down	CCL24[6]	5.8E1	0.0E0	n/d	0.020	10<br>1/3/5/1
		SNORA49	1.1E3	0.0E0	n/d	0.020	
		SNORD116-24	1.4E3	0.0E0	n/d	0.020	
		SNORD72	6.4E3	0.0E0	n/d	0.020	
		MIR301B	7.5E3	0.0E0	n/d	0.020	
		MIRLET7E	9.2E3	0.0E0	n/d	0.020	
		SNORD78	1.0E4	0.0E0	n/d	0.020	
		MIR32	6.4E4	0.0E0	n/d	0.020	
		TRNR[7]	1.0E7	1.8E5	-5.7	0.002	
II. Plasma	Up	MIR296	0.0E0	6.9E3	n/d	0.006	2<br>1/1/0/0
		METT5D1	3.4E1	8.3E3	7.9	0.041	
	Down	TRND[7]	3.0E5	0.0E0	n/d	0.027	61<br>17/3/26/15
		VTRNA1-1	5.3E4	0.0E0	n/d	0.006	
		SNORD81	5.2E4	0.0E0	n/d	0.018	
		SNORD38B	3.2E4	0.0E0	n/d	0.006	
		SNORD75	3.1E4	0.0E0	n/d	0.008	
		MIR3607	2.9E4	0.0E0	n/d	0.008	
		MIR3182	2.7E4	0.0E0	n/d	0.006	
		SNORD73A	2.7E4	0.0E0	n/d	0.016	
		SNORD2	2.4E4	0.0E0	n/d	0.006	
		SNORD38A	1.8E4	0.0E0	n/d	0.006	
		SNORD58B	1.8E4	0.0E0	n/d	0.006	
III. 16,000 g pellet	Up	MIR194-1	0.0E0	1.1E4	n/d	0.029	4<br>0/3/0/3
		MIR933	0.0E0	2.1E4	n/d	0.029	
		MIR4270	0.0E0	4.4E4	n/d	0.029	
		D-loop[7]	4.5E2	1.2E4	4.7	0.006	
	Down	RPS15A	4.7E1	0.0E0	n/d	0.046	4<br>1/1/0/2
		AluYc1	1.0E4	0.0E0	n/d	0.008	
		MIR4320	1.2E4	0.0E0	n/d	0.029	
		HSATII[8]	1.6E5	0.0E0	n/d	0.008	
IV. 160,000 g pellet	Up	SNORD113	0.0E0	2.7E3	n/d	0.041	4<br>0/1/2/1
		SNORD78	0.0E0	1.2E4	n/d	0.041	
		MIR520A	0.0E0	2.5E3	n/d	0.041	
		D-loop[7]	5.6E2	5.9E3	3.4	0.049	
	Down	DEFB109P1	2.0E2	0.0E0	n/d	0.019	12<br>1/7/2/2
		SNORA33	1.4E3	0.0E0	n/d	0.019	
		SNORD1B	2.8E3	0.0E0	n/d	0.019	
		MIR596	2.8E3	0.0E0	n/d	0.035	
		MIR3154	4.6E3	0.0E0	n/d	0.019	
		MIR134	5.4E3	0.0E0	n/d	0.019	
		MIR3124	6.9E3	0.0E0	n/d	0.019	
		MIR510	9.7E3	0.0E0	n/d	0.019	
		MIR409	9.8E3	0.0E0	n/d	0.019	
		MIR449B	3.8E4	0.0E0	n/d	0.019	
		c_tRNA-Pro[8]	4.0E5	0.0E0	n/d	0.016	
V. 160,000 g supernatant	Up	SNORD113	0.0E0	2.4E3	n/d	0.035	5<br>0/3/1/1
		MIR598	0.0E0	1.2E3	n/d	0.020	
		MIR642A	0.0E0	1.2E3	n/d	0.020	
		MIR1301	0.0E0	2.6E3	n/d	0.009	
		FLJ41278	3.1E1	5.3E4	10.7	0.04	
	Down	MIR374C	9.5E3	0.0E0	n/d	0.009	69<br>7/19/37/6
		SNORD50A	9.6E3	0.0E0	n/d	0.005	
		SNORD18A	1.0E4	0.0E0	n/d	0.012	
		MIR409	1.3E4	0.0E0	n/d	0.005	

Fraction	Regulation	Transcript ID	FPKM1[1] H	FPKM2[1] LSCC	log_2	q[2]	Total Number[3] NM/miR/sno/others[4]
		MIR376C	1.3E4	0.0E0	n/d	0.005	
		SNORD59B	2.0E4	0.0E0	n/d	0.005	
		SNORD48	2.6E4	0.0E0	n/d	0.005	
		SNORD81	4.9E4	0.0E0	n/d	0.012	
		SNORD29	5.9E4	0.0E0	n/d	0.005	
		TRND[7]	1.3E6	0.0E0	n/d	0.021	

(1) Human RNA profiles of healthy donors and patients with lung adenocarcinoma were compared fraction by fractions with Cuffdiff (cutoff 100). The most up regulated as well as the most down regulated RNA species for each pairs of comparisons are represented. Data sorted by log_2 in descending or ascending order for up or down regulated transcripts, respectively.
(2) Benjamini–Hochberg adjusted *p*-values of blood fractions FPKM values comparisons (CuffDiff). Benjamini–Hochberg-adjusted p-values of blood fractions FPKM values comparisons of healthy donor and lung adenocarcinoma patient blood fractions (Cuffdiff).
(3) Total number of differentially regulated RNAs for each block of up or down regulated species.
(4) Total number of mRNAs/microRNAs/snoRNAs/other ncRNAs (including lncRNAs, mtRNA, transcribed genome repeats rRNAs, tRNAs, U snRNAs, Y scRNAs) for the each block of up or down regulated species.
(5) For zero FPKM value in one of the compared fraction log2 value not determined (n/d). The data sorted by non zero FPKM value.
(6) For mRNAs with multiple splice isoforms the most exerted isoform was selected by log2 value of FPKM comparisons.
(7) Mitochondrial transcripts.
(8) Transcribed genome repeats.
